# Caring for trafficked and unidentified patients in the EHR shadows: Shining a light by sharing the data

**DOI:** 10.1371/journal.pone.0213766

**Published:** 2019-03-14

**Authors:** Sara H. Katsanis, Elaine Huang, Amanda Young, Victoria Grant, Elizabeth Warner, Sharon Larson, Jennifer K. Wagner

**Affiliations:** 1 Initiative for Science & Society, Duke University, Durham, North Carolina, United States of America; 2 Center for Translational Bioethics & Health Care Policy, Geisinger Health System, Danville, Pennsylvania, United States of America; 3 Center for Health Research, Geisinger Health System, Danville, Pennsylvania, United States of America; 4 Jefferson University College of Population Health, Philadelphia, Pennsylvania, United States of America; Florida International University, UNITED STATES

## Abstract

**Objective:**

Healthcare providers have key roles in the prevention of, detection of, and interventions for human trafficking. Yet caring for trafficked persons is particularly challenging: patients whose identities are unknown, unreliable, or false could receive subpar care from providers delivering care in a vacuum of relevant information. The application of precision medicine principles and integration of biometric data (including genetic information) could facilitate patient identification, enable longitudinal medical records, and improve continuity and quality of care for this vulnerable patient population. Scant empirical data exist regarding healthcare system preparedness and care for the needs of this vulnerable population nor data on perspectives on the use and risks of biometrics or genetic information for trafficked patients.

**Methods:**

To address this gap, we conducted mixed-methods research involving semi-structured interviews with key informants, which informed a subsequent broad survey of physicians and registered nurses.

**Results:**

Our findings support the perception that trafficked persons obtain care yet remain unnoticed or undocumented in the electronic health record. Our survey findings further reveal that healthcare providers remain largely unaware of human trafficking issues and are inadequately prepared to provide patient-centered care for trafficked and unidentified patients.

**Conclusion:**

Meaningful efforts to design and implement precision medicine initiatives in an inclusive way that optimizes impacts are unlikely to succeed without concurrent efforts to increase general awareness of and preparedness to care for trafficked persons. Additional research is needed to examine properly the potential utility for biometrics to improve the delivery of care for trafficked patients.

## Introduction

Over the past decade, scientists have increasingly placed faith (and resources) in the potential for genomic science and health information technologies to transform medicine from a reactive endeavor to a proactive, anticipatory one [1–6]. A more holistic approach to preventing, diagnosing, and treating diseases as well as promoting health relies heavily upon the integration of large amounts of data from clinical and non-clinical sources and the responsible sharing of those data broadly in order to maximize the potential insights (for individuals and populations) that can be gleaned. While genomics and health information technologies offer great promise to improve health care and advance science, there are legitimate concerns that everyone will not share equitably in the process or the progress (e.g., [7–13]). Historically recognized and emerging vulnerable populations require specific attention as health systems implement the components for precision medicine in order to mitigate known health disparities and prevent their exacerbation. With appropriate attention during the design stages and regular critical assessment during implementation stages, precision medicine efforts could achieve the desired human-centered process and outcomes. Among the vulnerable populations deserving of attention are trafficked persons, for whom the net benefits of the application of precision medicine principles and use of biometric data could be substantial and for whom the ethical, legal, and social implications of such applications of biometric data might necessitate special procedural and/or substantive considerations to prevent harm. Risks to using biometric data including genetic information in the EHR might include (a) reduced personal or familial privacy; (b) reduced trust in healthcare professionals and institutions; (c) secondary uses of data by law enforcement or government entities; and (d) the unintentional disclosure of unknown or hidden relationships detected through biometric data; whereby the benefit of using biometrics is in the ability to connect medical records for individuals and thereby provide continuity of care to a population that frequents hospital systems and might use false identities upon admission.

Human trafficking (HT), a form of modern slavery, is a serious public health problem [14]. HT occurs in all 50 U.S. states and includes both labor and sex trafficking [15]. Pennsylvania, New Jersey, and North Carolina (the geographic area served by the study sites) are recognized source, transit, and destination states for HT, having ranked in 2015 among the top 12 states for number of calls to the National Human Trafficking Hotline [16] and the top 15 states for reported HT cases [17]. Scholars have estimated 37–50% of trafficked persons encounter healthcare professionals during their captivity [18], with one extensively cited report finding as many as 88% of trafficked persons having contact with a healthcare provider during their exploitation ([19]; see also [18, 20–21]). Ongoing efforts aim to improve the training of healthcare professionals to recognize indicators of HT [22–24], improve the identification of trafficked persons [25], apply victim-centered and trauma-informed care consistently [26], improve healthcare system responses to HT [22, 27], and develop specific materials for healthcare settings [28–29]. However, until fiscal year 2019 [30], there were no ICD Codes specific for suspected human trafficking [31–32], making it difficult for providers to document suspected cases of HT or ensure continuity of care for trafficked persons. Recognized complications of healthcare for trafficked individuals include (a) fragmented documentation of medical history across multiple healthcare systems; (b) lack of candid communication with healthcare providers and; (c) presence of an oppressor who might accompany patients to clinic appointments.

A host of factors can explain why interaction between trafficked persons and the healthcare system often goes undetected by healthcare providers. Identifying a patient as a trafficked person requires providers to adapt a perceptive eye for cues potentially falling outside what they perceive to be the purview of daily medical practice. Defining what constitutes HT is a contentious topic and varies substantially depending upon a person’s background, biases, and experience with HT. For instance, women who work as sex workers often are vilified and stereotyped in Western society [33]. Additionally, the legal definition of HT can vary across jurisdictions in its inclusion of arranged child marriages, consensual sex work, and organ trafficking [34] (S1 Appendix for definitions [35–36]). Other factors—such as a language barrier, a close (and, perhaps, even conspiring) relationship between the trafficker and healthcare provider, and a victim’s shame and guilt—might either prevent healthcare providers from realizing they are treating a trafficked person or preclude any documentation or reporting of the patient’s known or suspected trafficked status [18]. Because encountering a trafficked person is not a common situation for which healthcare providers can readily share similar experiences, lack of awareness of trafficking in the healthcare setting and “Groupthink” might also impede an appropriate healthcare response to HT, leading to complacency and rationalized inaction [37].

While educational interventions have been shown to improve healthcare providers’ ability to recognize trafficked persons [38], there is a dearth of empirical evidence to support the identification and care of trafficked persons [39]. HT education offered for healthcare providers varies widely, and programs are rarely evaluated for effectiveness [40]. Healthcare screening tools for trafficked persons are similarly lacking in demonstrated effectiveness and varying in content and length [41]. Standardization of screening tools and identification practices based on empirically validated research is necessary not only to ensure appropriate methods of intervention that lead to optimal patient outcomes but also to provide continuity of care to the patients who are trafficked persons.

Along with training healthcare providers in best practices for recognizing a trafficked person and managing their care, biometric technologies (e.g., palm readers; fingerprinting; iris scans; and genetic data) have the potential to aid in the identification of and continuity of care for trafficked persons [42]. Healthcare systems have implemented fingerprint scanning to reduce insurance-card fraud, accurately distinguish patients with similar names or birthdates, and link patient data across various healthcare institutions, thus allowing the electronic health record (EHR) data to travel with the patient instead of being confined to a single clinic site or single healthcare system [43]. Biometrics could detect cases of misidentification and false identity—which might be common for trafficked persons. Whether or not such a patient is a trafficked person would be a distinct question altogether, and that question would be one better assessed by traditional, non-biometric evaluations. Nevertheless, identifying that the same patient has made multiple visits to a clinic can help in provision of care by giving them a better understanding of that patient’s medical history. A healthcare provider with such knowledge of the patient’s inconsistent presentations of identity and longitudinal health records would be empowered to then engage in trauma-informed, patient-centered care even if the patient were accompanied in the clinic by a controlling person. In many HT cases, the trafficked person is unwilling to seek assistance to escape their situation, much like a domestic abuse case. Yet our prior work has shown that trafficked persons trust healthcare providers more than many other authorities [44]. In other cases, a person entering the healthcare system unidentified and unable to supply identification (e.g., unresponsive, comatose, or even deceased) might be a trafficked person. Determining a person’s identity is valuable for care and, sometimes, essential for successful prosecution of crimes causing their unidentified patient state.

Despite the growing recognition that healthcare professionals must be part of a comprehensive solution to HT problems and despite the growing recognition that engagement of trafficked persons is wrought with challenges requiring flexible care approaches [45], there are scant empirical data regarding healthcare system preparedness for the specific patient care needs of this population. Furthermore, the time is ripe to consider how the routinization of genomic technologies and broad data sharing within and across health systems (spurred by precision medicine purposes) could uniquely affect this vulnerable population. Unfortunately, to date there have been no known studies exploring the use of biometrics generally or genetic information specifically to assist in the identification of trafficked persons or for continuity of care for unidentified patients. To address this gap, we embarked on an early exploration of these challenging and ethically-charged questions by conducting mixed-methods research at and beyond Geisinger Health System.

## Methods

The main motivation for this study was to investigate applications and implications for precision medicine tools and principles—specifically genetic information, health information technologies and integration of data from clinic and non-clinic sources, and data sharing issues within and across health systems—as they relate to healthcare delivery for a specific vulnerable population: trafficked and unidentified patients. The study design was developed with a mixed-methods approach with three components. The first component was a preparatory-to-research EHR data pull (*i*.*e*., a non-hypothesis-testing review of EHR data) facilitated by a data broker to gauge informally, through participant observation, the feasibility of conducting HT research using data readily available from the EHR. The second component involved a series of key informant interviews of stakeholders from diverse healthcare settings (an integrated health system; an independent community hospital; and an academic medical center) to explore perspectives about HT as a public health problem, learn operational details regarding the current delivery of healthcare for trafficked and unidentified patients and perceived barriers to care, and elicit opinions (including individual, institutional, societal, legal, and scientific/technical issues) regarding biometrics and data sharing efforts intended to improve identification and continuity of care for this vulnerable population. The key informant interviews also were intended to provide an opportunity to pilot, validate questions, and inform a survey instrument for use as the third component of this study: a broad survey of physicians and nurses. We elaborate on each mixed-methods component below.

### EHR data pull

A preliminary examination of system-wide EHR data was performed by a data broker in November 2016 to better understand the scale with which one healthcare system encounters potential trafficked persons or the unidentified as patients. The purpose of this review was not to test any scientific hypotheses or examine individual patient charts; rather, the purpose was to engage in participant observation that would enable the research team to understand the difficulties in gathering relevant data from the EHR for HT-related public health research. Of interest was the number of patients encountered system-wide and by clinic location who might be trafficked persons or unidentified, which would be foundational information needed if investigators wanted to design robust public health research studies with sufficient statistical power and appropriate sampling frames.

Geisinger was an ideal study site given its broad reach across two states, connectivity via a common EHR, progressive development of precision medicine tools, and experienced data brokers (S2 Appendix for further explanation). The study team (see S3 Appendix) developed an initial set of variables to be searched by the data brokers based on information from the literature. The search was time-delimited, querying records from 2000 to 2016. Variables used to identify potential cases included a code rumored to be in use (i.e., “Trauma 181” code in the name field); identity unknown at admission to an emergency department; fake identification used; adult individuals appearing with a controlling companion; low utilizers who first appear to an OB/GYN or prenatal clinic late in pregnancy; those who have sexual abuse confirmed or suspected; or those malnourished. To narrow these broad search variables, the data broker also queried whether there was a known address or indication of a non-English first language. Other variables included whether minors were admitted to the emergency department without an adult present. The data broker revealed only counts of potential cases to the study team, and no chart reviews were performed.

The preparatory-to-research EHR data pull request at Geisinger was for counts across the entire system and from different clinic locations, but the data broker was able to report only on a system level because of the limited findings. Several prioritized variables were unsuccessful: no records were located for the particular codes searched, including (a) identity unknown at admission to emergency room, (b) fake identification used, or (c) adult individuals who appear with a controlling companion (Table 1).

**Table 1:** Geisinger EHR system variable requests and results from data broker.

Record Request	Total Number Hits	Without Address	Non-English Speakers
“Trauma 181” code	-	-	-
Identity unknown at admission to emergency	-	-	-
Fake identification used	-	-	-
No primary care visits, more than 1 ER visit	2,836	1	71
First OB/GYN or prenatal clinic visit in 3^rd^ trimester	2,077	1	268
Sexual abuse confirmed or suspected*	126	2	5
Malnutrition**	6,044	4	509
Minor emergency admission without an adult	-	-	-
Minor with sexual abuse confirmed or suspected***	1,645	5	126

* ICD-9 code 995.83 or ICD-10 code T74.21XA

**ICD-9 code 263 or ICD-10 code 44

***ICD-10 code T74.22xa or T76.22xa

The study team observed first-hand difficulties that public health researchers face when attempting to initiate research regarding delivery of care for trafficked persons. Variables inferred from the literature to identify the target population for the research (from which robust study designs could be developed—regardless of whether those studies were focused on interactions with the trafficked patients or the physicians, nurses, and other professionals delivering their care) were concurrently over- and under-inclusive, lacking both specificity and sensitivity to generate useful information. Studying HT through data readily available in the EHR without HT-specific ICD codes was all but impossible. Based on this participant observation, the study team developed the interview guide and approach for the second component of the study and decided not to focus efforts on a particular geographic location or on a particular department or specialty area for healthcare providers at the study sites.

### Informant interviews

We developed a semi-structured interview guide (S1 Instrument) to explore (A) general preparedness of healthcare professionals in meeting the needs of this patient population and (B) patient-centered, sustainable, and effective approaches for caring for trafficked persons and unidentified patients. This approach was elected to gather preliminary themes on the challenges facing healthcare providers in the early implementation of HT screening tools and biometrics for identification of trafficked persons. Interviews were conducted in an integrated health system in Pennsylvania (Geisinger, PA), an independent community hospital in the same geographic region (Evangelical Community Hospital, PA), and at a large academic medical center in North Carolina (Duke University, NC) to gather both common and differentiating trends among these three types of healthcare-providing organizations. Prospective informants were invited to participate in semi-structured interviews. Potential Pennsylvania informants included physicians, nurses, administrators, and other healthcare staff identified as having some relevant specialty or administrative expertise or identified through snowball sampling. Potential North Carolina informants included Duke physicians in emergency and OB/GYN selected through random sampling. Interviews were conducted by phone during the summer of 2017 and audio-recorded to enable transcription. Informants were asked to provide sociodemographic information and to complete a pilot survey. Recordings were transcribed to enable qualitative content and theme analysis. Two investigators independently reviewed the transcripts to define emerging themes and corresponding quotes across all participants. Themes were identified by consensus across interviews and topics with contrasting viewpoints and then grouped into broader categories for coding. Investigators then applied the themes to the quotes to select illustrative perspectives.

### Survey of physicians and registered nurses

Based upon information learned during the first two components of this mixed-methods study, we developed a survey of physicians and registered nurses regardless of geographic location, specific department, or care specialty. The pilot survey used during the key informant interviews, which was initially designed by incorporating important items drawn from the literature (e.g., [46]), was refined and subsequently administered throughout Geisinger Health System using Survey Monkey (www.surveymonkey.com; Palo Alto, CA; See S2 Instrument). Survey items related specifically to biometrics (such as genetic data) and precision medicine principles (such as data sharing) were kept to a minimum, as the discussion seemed premature given the findings of the key informant interviews. The survey contained 33 items. Recruitment messages were sent to all physicians and registered nurses by email as “Dear Colleague” messages from the study’s principal investigator, and a follow-up recruitment message was sent after one week. The collector was open for two weeks, and no research incentive was provided. A total of 5875 healthcare providers (comprising 1517 physicians and 4358 nurses) received the recruitment message. Statistical analyses were performed using SAS 9.4 (SAS Institute, Inc., Cary, NC). Comparisons between categories were tested using chi-square tests or the Fisher’s exact test depending upon the number of categories compared.

### Ethics approval

This mixed-methods research was conducted pursuant to an IRB exemption determination from Geisinger IRB (#2017–0319). Implied informed consent was provided by key informants and survey respondents through their participation in the interviews and/or survey. Permission to report the preparatory-to-research data broker findings was obtained separately (#2017–0182). No institution or funding source had any involvement in this study’s design; collection, analysis, or interpretation of data; or decision to publish.

## Results

### Informant interviews

Nine (N = 9) informant interviews were conducted before data saturation was reached. In Pennsylvania thirty-five (35) prospective participants (including departmental chairs, physicians, nurses, and administrators in emergency/urgent care, OB/GYN, and psychiatry in nine locations) were contacted with six (6) agreeing to interviews. In North Carolina, sixteen (16) physicians were contacted (half OB/GYN, half emergency), with three (3) agreeing to interviews. The overall response rate was 18% (9/51). Interviewees’ demographics are reported in Table 2. While a diverse group of providers were contacted, interviewees were only White and heterosexual. In North Carolina no emergency physicians were interviewed, and in Pennsylvania no OB/GYN physicians were interviewed. Interviewees were asked about (1) their experiences with and understanding of HT and (2) their knowledge of how the health system manages HT cases and unidentified persons to address HT identification and continuity of care. We identified eight themes. Selected illustrative quotations are shown in Table 3.

**Table 2:** Demographic characteristics of interview participants.

Participant	Specialty	Role	Age	Sex	Surroundings
NC1	OB/GYN	Physician	56–65	M	Urban
NC2	OB/GYN	Physician	56–65	F	Suburban
NC3	OB/GYN	Physician	46–55	F	Urban
PA1	Psychiatry	Nurse	46–55	F	Rural
PA2	Psychiatry	Administrator	56–65	M	Rural
PA3	Emergency	Physician	46–55	M	Suburban
PA4	Emergency	Nurse	46–55	F	Rural
PA5	Emergency	Nurse	36–45	F	Rural
PA6	System-wide	Administrator	56–65	F	*Not provided*

**Table 3:** Selected interviewee quotations.

Observation	Illustrative Quotations from Informants
**Healthcare providers would like to see improved policy and care guidelines for managing trafficked patients, including overcoming biases and minimizing stigmatization.**	“I don’t know how much awareness there is … healthcare providers sometimes, when we see these people that come in from different areas, when you have high drug abuse, high violence–whether it be physical violence [or] sexual assault–with a lot of behavioral health disorders and stuff like that. Sometimes, we don't always look for those signs [of HT]. …It doesn’t happen overnight.” (PA4)
	“we shouldn't be labeling them, we should be reaching out to try to help them, so giving them a specific ICD-10 code for a trafficking victim–when that's not really what they're seeking care for–we should be identifying what they're seeking care for and then identify that they are a trafficking victim and what help [they need], not give them a label.” (PA5)
**There is disagreement as to whether or not biometrics is appropriate for HT patient care management.**	“we could move in that direction with patients, whether it's fingerprints or eye scans or any number of unique identifiers to help us make sure we absolutely have the right patient” (NC2)
	“Some kind of biometric identification would be ideal, whether it's a palm scanner, which the system has talked about previously, or retinal scanning, or anything like that” (PA3)
	“I do think that would be extremely helpful” (PA4)
	“I think it would help. You would have a continuous chart.” (NC3)
	“I would think that … the idea that there could be fingerprinting or anything like that would make people shy away from coming to a big [healthcare] system [like ours]” (PA1)
	“There are specific places that are using voice recognition, but it's tough if you have an unconscious patient” (PA4)
	“I'm not sure if it would be helpful or if it would be felt as being invasive” (PA5)
	“I think that might increase paranoia a little bit” (NC2)
	“It's not going to help you if they have none [no biometrics in the system already] and they haven't been here before” (PA6)
	“[Biometrics are] not really utilized in the medical care of the patient. I'm not really sure how I would use that” (NC1)

#### 1. Interviewees view “human trafficking” as a broad term

Several interviewees used common terms including “forced” and “unwilling” to describe how they would define HT. Seven mentioned sex trafficking in particular, and three referenced minors as at-risk. One (PA3) seemed familiar with the TVPA definition, if not citing it precisely. Responses varied widely regarding where HT victims originate, with five acknowledging trafficked persons can come from “all over” or “anywhere” and four interviewees identifying specific geographic regions (including Mexico, Asia, or South America).

#### 2. HT was rarely encountered among providers

Six interviewees agreed that HT exists in their geographic area, but only two (PA2 & NC2) reported having encountered it personally. Three interviewees (PA1, PA3, and NC3) did not think that HT happened in their geographic area. Most interviewees acknowledged if a trafficked person presented to the hospital he/she might be seen by any level of care provider, including physicians, nurses, front desk staff, and EMT. Two interviewees highlighted the importance nurses might play in building patient trust (NC2 and PA3). When asked which specialty area is likely to encounter trafficked persons, interviewees cited emergency management, OB/GYN, psychiatry, and pediatrics. When asked hypothetically how they would proceed if they encountered a suspected case of HT as a provider, interviewees cited the use of a special care management team (such as social workers); the need to confirm the patient had a “safe place”; and the need to involve the police or do mandatory reporting in cases that involved children. At least one provider (NC2) expressed concern that reporting a suspected case of HT to law enforcement meant the patient might not return to their care. Several likened care management plans for trafficked patients to those used for domestic abuse patients.

#### 3. Interviewees were unaware of institutional resources or policies specific for care of trafficked persons

One interviewee (PA5) indicated awareness of early stages of development of policies and procedures for managing care for trafficked persons in an emergency department. Others, while personally knowledgeable about specific care needs of trafficked persons, were unaware of any institutional policy defining HT or stipulating any care management protocol. None were aware of EHR codes for specific documentation of a person’s trafficked status, although several indicated that there probably were codes that they did not know about personally.

#### 4. Healthcare providers would like to see improved policy and care guidelines for managing trafficked patients

Several interviewees indicated a need for educational tools, including “training of providers to recognize potential signs” and “community engagement” (PA2); “a fact sheet of what to do and what is allowable by law and what isn’t” (PA3); “continuing education program, which might increase awareness” (NC1); and “more information on what the local resources are for [providers]” (NC3). One provider (NC2) suggested a barrier was the lack of privacy to talk with trafficked patients that might be accompanied by the trafficker, suggesting that the clinics have “more privacy in the intake area,” and “more confidential, encased spots where [the providers] are truly alone with the patients come in.” One interviewee (PA4) suggested that overcoming bias (both implicit and explicit) of patients who might be trafficked persons was an issue, and another (PA5) discussed how provision of appropriate healthcare for trafficked persons is most needed rather than documentation of trafficked status.

#### 5. The scope of types of unidentified patients seen in healthcare is broad and might include trafficked persons

Interviewees discussed two broad categories of unidentified patients: (1) those who are incapable of providing identification due to their physical state (e.g., unconscious patients) and (2) those who choose to provide false identification (e.g., victims of domestic abuse trying to remain hidden). The interviewees reported common management plans including assigning patients a number or “John/Jane Doe” names in their medical record for documenting the uncertainty of the patient’s identity. Interviewees had not considered whether unidentified persons might need to be screened for HT.

#### 6. Limited medical record tools and EHR codes are currently in use for tracing continuity of care

Interviewees in both states discussed existing medical record tools that could be harnessed for documenting patients that might be trafficked persons, such as the rumored “trauma 181” code at Geisinger (assessed for the EHR data pull) and domestic abuse codes. Similarly, interviewees in Pennsylvania described the tools for unidentified patients that might be harnessed to help with continuity of care for HT victims. They specifically described a recycling numbering method for labelling unidentified patients (e.g., Patient 1, 2, 3, … 199, 200, 201, …); however, no interviewees acknowledged having used these codes for documenting a person who might have been trafficked.

#### 7. Biometrics is currently in use in some areas of patient management

Interviewees mentioned use of biometrics in contexts outside of HT, and one mentioned its possible utility for tracing unconscious patients. PA6 mentioned efforts to incorporate palm scan protocols at intake for healthcare management of patients who have identical or similar names. PA4 mentioned having heard of clinics using voice recognition software for patient identification. NC3 mentioned awareness of the use of retinal scans in clinics to minimize medical error. No interviewees were aware of the use of biometrics for unidentified patients or for streamlining continuity of care.

#### 8. There is disagreement as to whether or not biometrics is appropriate for HT patient care management

Interviewees expressed a broad spectrum of reactions to the idea of using biometrics for patient identification. Several recognized the value of biometrics as part of a medical record in maintaining continuity of care, but many acknowledged biometrics could be perceived as overly invasive. Two had an understanding of biometrics that did not encompass continuity of care capabilities.

### Survey responses from physicians and registered nurses

**Fig 1:**
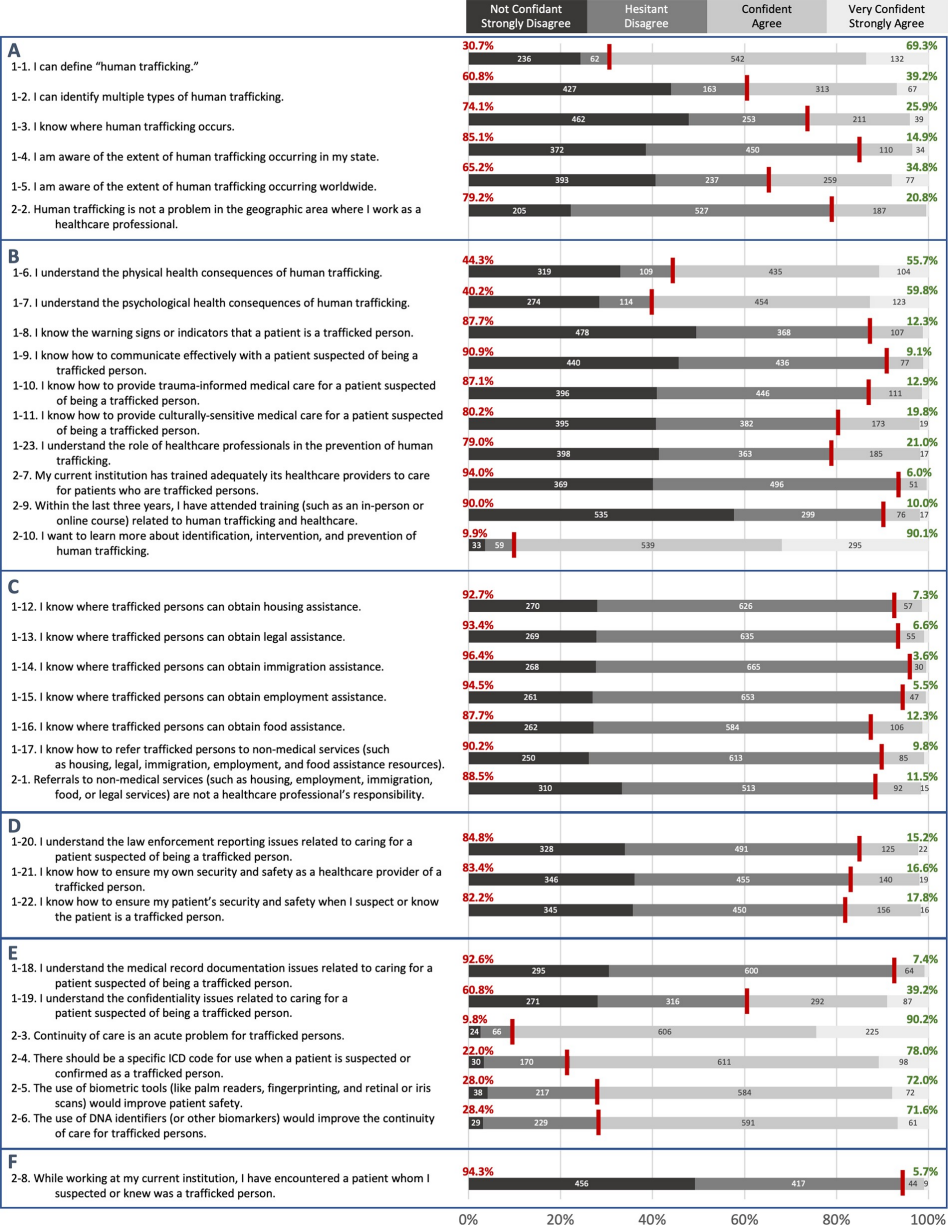
Agreement and confidence of respondents. Participants were asked via survey to assess their confidence in a set of statements and to assess their agreement to a second set of statements. Statements numbered “1-**” were hesitant/confident formatted and questions numbered “2-**” were disagree/agree formatted. Non-respondents are not included in percentage calculations, which comprise no more than 6.7% of respondents. A) Respondents demonstrated confidence in defining human trafficking, but less confidence in their understandings of the extent of trafficking. Most agreed that human trafficking was a problem in their geographic area. B) While just over half of respondents claimed to understand the physical and psychological health consequences that present in trafficking victims, the majority of respondents were not confident in their knowledge of how to provide care to trafficked persons. A strong majority of respondents did not feel that they had been trained adequately and want to learn more about human trafficking. C) Respondents were not confident in their ability to provide non-medical assistance to trafficked persons and a strong majority agree that referrals to non-medical services is their responsibility.* D) The majority of respondents were not confident in their understanding of how to report trafficking and ensure safety of trafficked patients and themselves. E) A majority of respondents were not confident in the issues of medical record documentation and the confidentiality issues for trafficked persons. However, a strong majority did see continuity of care to be an acute problem for trafficked persons. In addition, a majority of respondents indicated that using ICD codes for trafficked persons and biometrics, including DNA, could be used to trace patients that are trafficked. F) Almost all of these responses are hypothetical, as the majority of respondents have not knowingly encountered a patient that was a trafficked person. ^*^Q2-1 was phrased as a double-negative, *which may have affected the responses*.

Survey responses were collected in December 2017 from 972 respondents for a response rate of 16.5%. The complete survey statements and responses are shown in Table 4 and illustrated in Fig 1. Respondents consisted of 162 physicians and 738 registered nurses, with most identifying as women (75.8%); as White, European American, or European (83.1%); and as residing in rural areas (63.4%) (see Table 5 for respondents’ demographic characteristics). While two-thirds of respondents indicated confidence in their ability to define “human trafficking” (69.4%), only one-eighth (12.2%) reported an awareness of the warning signs or indicators that a patient is a trafficked person. Less than one-fifth indicated confidence in their abilities to provide trauma-informed (12.8%) and culturally sensitive care (19.8%) for patients who are trafficked persons. Respondents overwhelmingly (>85%) reported little or no confidence in their knowledge about where trafficked persons might obtain non-medical assistance (such as housing, legal, immigration, employment, and food assistance) and similarly reported lack of confidence in how to refer patients to such resources. Only 53 respondents (5.4%) reported they encountered a patient suspected or known to be a trafficked person. While a majority of respondents (55.3%) indicated their belief that human trafficking is not a problem in the geographic area where they work, an even greater majority (89.0%) indicated their current institution has not adequately trained its providers to care for such patients. Few (9.6%) reported having attended recent training relevant to the topic. Respondents expressed considerable interest (85.9%) in learning more about the identification, intervention, and prevention of HT. There was general agreement that continuity of care is an acute problem for trafficked persons (85.6%), that a specific ICD code should be available for use for when a patient is suspected or confirmed as a trafficked person (73.0%), and that biometric tools could improve both patient safety (67.5%) and continuity of care for trafficked persons (67.1%).

**Table 4:** Survey responses on perspectives of physicians and registered nurses. **A.** Responses to statements reflecting awareness and understanding of human trafficking, and preparedness for encountering a human trafficking case; **B.** Responses to statements reflecting perspectives on relevant concepts. **N (%)**. NR = non-response; Statistically significant differences occurring by professional role (P), department (D), years (in other words, tenure) in the profession (T), race (R), and gender identity (G) of respondent are displayed, where appropriate, in the right-most column.

AWARENESS, UNDERSTANDING AND PREPAREDNESS	Hesitant	Not Confident	Confident	Very Confident	NR	Notable Differences
*1*. *I can define “human trafficking*.*”*	236 (24.3)	62 (6.4)	542 (55.8)	132 (13.6)	0 (0.0)	
*2*. *I can identify multiple types of human trafficking*.	427 (43.9)	163 (16.8)	313 (32.2)	67 (6.9)	2 (0.2)	
*3*. *I know where human trafficking occurs*.	462 (47.5)	253 (26.0)	211 (21.7)	39 (4.0)	7 (0.7)	T
*4*. *I am aware of the extent of human trafficking occurring in my state*.	372 (38.3)	450 (46.3)	110 (11.3)	34 (3.5)	6 (0.6)	
*5*. *I am aware of the extent of human trafficking occurring worldwide*.	393 (40.4)	237 (24.4)	259 (26.7)	77 (7.9)	6 (0.6)	D
*6*. *I understand the physical health consequences of human trafficking*.	319 (32.8)	109 (11.2)	435 (44.8)	104 (10.7)	5 (0.5)	
*7*. *I understand the psychological health consequences of human trafficking*.	274 (28.2)	114 (11.7)	454 (46.7)	123 (12.7)	7 (0.7)	
*8*. *I know the warning signs or indicators that a patient is a trafficked person*.	478 (49.2)	368 (37.9)	107 (11.0)	12 (1.2)	7 (0.7)	
*9*. *I know how to communicate effectively with a patient suspected of being a trafficked person*.	440 (45.3)	436 (44.9)	77 (7.9)	11 (1.1)	8 (0.8)	
*10*. *I know how to provide trauma-informed medical care for a patient suspected of being a trafficked person*.	396 (40.7)	446 (45.9)	111 (11.4)	14 (1.4)	5 (0.5)	D
*11*. *I know how to provide culturally-sensitive medical care for a patient suspected of being a trafficked person*.	395 (40.6)	382 (39.3)	173 (17.8)	19 (2.0)	3 (0.3)	T
*12*. *I know where trafficked persons can obtain housing assistance*.	270 (27.8)	626 (64.4)	57 (5.9)	14 (1.4)	5 (0.5)	
*13*. *I know where trafficked persons can obtain legal assistance*.	269 (27.7)	635 (65.3)	55 (5.7)	9 (0.9)	4 (0.4)	G
*14*. *I know where trafficked persons can obtain immigration assistance*.	268 (27.6)	665 (68.4)	30 (3.1)	5 (0.5)	4 (0.4)	
*15*. *I know where trafficked persons can obtain employment assistance*.	261 (26.9)	653 (67.2)	47 (4.8)	6 (0.6)	5 (0.5)	
*16*. *I know where trafficked persons can obtain food assistance*.	262 (27.0)	584 (60.1)	106 (10.9)	13 (1.3)	7 (0.7)	
*17*. *I know how to refer trafficked persons to non-medical services (such as housing*, *legal*, *immigration*, *employment*, *and food assistance resources)*.	250 (25.7)	613 (63.1)	85 (8.7)	9 (0.9)	15 (1.5)	
*18*. *I understand the medical record documentation issues related to caring for a patient suspected of being a trafficked person*.	295 (30.4)	600 (61.7)	64 (6.6)	8 (0.8)	5 (0.5)	
*19*. *I understand the confidentiality issues related to caring for a patient suspected of being a trafficked person*.	271 (27.9)	316 (32.5)	292 (30.0)	87 (9.0)	6 (0.6)	P, D
*20*. *I understand the law enforcement reporting issues related to caring for a patient suspected of being a trafficked person*.	328 (33.7)	491 (50.5)	125 (12.9)	22 (2.3)	6 (0.6)	
*21*. *I know how to ensure my own security and safety as a healthcare provider of a trafficked person*.	346 (35.6)	455 (46.8)	140 (14.4)	19 (2.0)	12 (1.2)	
*22*. *I know how to ensure my patient’s security and safety when I suspect or know the patient is a trafficked person*.	345 (35.5)	450 (46.3)	156 (16.1)	16 (1.7)	5 (0.5)	D
*23*. *I understand the role of healthcare professionals in the prevention of human trafficking*.	398 (41.0)	363 (37.4)	185 (19.0)	17 (1.8)	9 (0.9)	T
**PERSPECTIVES**	**Strongly Disagree**	**Disagree**	**Agree**	**Strongly Agree**	**NR**	**Notable Differences**
*24*. *Referrals to non-medical services (such as housing*, *employment*,* immigration*, *food*, *or legal services) are not a healthcare professional’s responsibility*.	310 (31.9)	513 (52.8)	92 (9.5)	15 (1.5)	42 (4.3)	P, R, G
*25*. *Human trafficking is not a problem in the geographic area where I work as a healthcare professional*.	205 (21.1)	527 (54.2)	187 (19.2)	5 (0.5)	48 (4.9)	G
*26*. *Continuity of care is an acute problem for trafficked persons*.	24 (2.5)	66 (6.8)	606 (62.4)	225 (23.2)	51 (5.3)	P, T
*27*. *There should be a specific ICD code for use when a patient is suspected or confirmed as a trafficked person*.	30 (3.1)	170 (17.5)	611 (62.9)	98 (10.1)	63 (6.5)	D
*28*. *The use of biometric tools (like palm readers*, *fingerprinting*, *and retinal or iris scans) would improve patient safety*.	38 (3.9)	217 (22.3)	584 (60.1)	72 (7.4)	61 (6.3)	
*29*. *The use of DNA identifiers (or other biomarkers) would improve the continuity of care for trafficked persons*.	29 (3.0)	229 (23.6)	591 (60.8)	61 (6.3)	62 (6.4)	
*30*. *My current institution has trained adequately its healthcare providers to care for patients who are trafficked persons*.	369 (38.0)	496 (51.0)	51 (5.3)	4 (0.4)	52 (5.4)	P, G
*31*. *While working at my current institution*, *I have encountered a patient whom I suspected or knew was a trafficked person*.	456 (46.9)	417 (42.9)	44 (4.5)	9 (0.9)	46 (4.7)	
*32*. *Within the last three years*, *I have attended training (such as an in-person or online course) related to human trafficking and healthcare*.	535 (55.0)	299 (30.8)	76 (7.8)	17 (1.8)	45 (4.6)	D
*33*. *I want to learn more about identification*, *intervention*, *and prevention of human trafficking*.	33 (3.4)	59 (6.1)	539 (55.5)	295 (30.4)	46 (4.7)	P, G

**Table 5:** Demographic characteristics of survey respondents. NR = Item non-response.

	N (%)
**Role as a Healthcare Professional**	
Physician	162 (16.7)
Nurse	738 (75.9)
Other	15 (1.5)
Prefer not to answer	6 (0.62)
NR	51 (5.2)
**Primary Department**	
Emergency	74 (7.6)
OB/GYN	50 (5.1)
Psychiatry	26 (2.7)
Pediatrics	68 (7.0)
Other	634 (65.2)
Prefer not to answer.	40 (4.1)
NR	80 (8.2)
**Primary Work Zip Code**	
166**	3 (0.31)
168**	21 (2.2)
170**	169 (17.4)
171**	6 (0.62)
177**	12 (1.2)
178**	426 (43.8)
179**	14 (1.4)
180**	1 (0.10)
184**	9 (0.93)
185**	57 (5.9)
186**	11 (1.13)
187**	123 (12.7)
NR	120 (12.3)
**Years in the Healthcare Profession**	
Fewer than 10 years	281 (28.9)
10–19 years	214 (22.0)
20–29 years	170 (17.5)
30 years or more	240 (24.7)
Prefer not to answer.	15 (1.5)
NR	52 (5.4)
**Age**	
18 to 25 years old	58 (6.0)
26 to 35 years old	211 (21.7)
36 to 45 years old	184 (18.9)
46 to 55 years old	209 (21.5)
56 to 65 years old	217 (22.3)
66 to 75 years old	22 (2.3)
Prefer not to answer.	16 (1.7)
NR	55 (5.7)
**Educational Attainment**	
Grade 12 or GED (high school graduate)	1 (0.1)
1 to 3 years after high school (some college, Associate's degree, or technical school)	223 (22.9)
College 4 years or more (college graduate)	434 (44.7)
Advanced degree (Master's, Doctorate, etc.)	245 (25.2)
Prefer not to answer.	15 (1.5)
NR	54 (5.6)
**Residential Characteristics**	
Rural	616 (63.4)
Suburban	247 (25.4)
Urban	42 (4.3)
Prefer not to answer.	17 (1.8)
NR	50 (5.1)
**Place of Birth**	
In the United States	866 (89.1)
Outside of the United States	49 (5.0)
Prefer not to answer	6 (0.6)
NR	51 (5.3)
**Race and ethnicity**	
White, European American, or European	808 (83.1)
American Indian or Alaska Native	1
Asian	30
Black, African American, or African	8
Hispanic, Latino, or Spanish	12
Middle Eastern or North African	3
Native Hawaiian or other Pacific Islander	1
None of these fully describe me.	19
Prefer not to answer.	40 (4.1)
NR	50 (5.1)
**Gender identity**	
Woman	737 (75.8)
Man	166 (17.1)
None of these fully describe me.	3 (0.3)
Prefer not to answer.	15 (1.5)
NR	51 (5.3)

Responses varying significantly by professional role, department, years in the profession (i.e., tenure), race, and gender identity are summarized in the right-most column of Table 4. Physicians and nurses were similar in their reported confidence in their ability, understanding, and preparedness to care for patients who are trafficked persons (S1 Table) with few exceptions. They differed in ability to understand the confidentiality issues related to caring for a patient suspected of being a trafficked person and in perspectives regarding continuity of care as an acute problem for trafficked persons, adequacy of the training at their institution, and desire to learn more about HT. There were no differences in responses among respondents who reside in rural areas versus those who reside in less rural areas (S2 Table). White, European American, and European respondents when compared to all others showed a significant difference in their levels of agreement that there should be an ICD code for suspected or confirmed trafficking and their perspectives regarding adequacy of the training at their institution (S3 Table). Significant differences among gender identity were found regarding belief that HT is not a problem in the geographic area, adequacy of the training at their institution, and desire to learn more about HT (S4 Table). Some aspects of ability, understanding, and preparedness differed by healthcare department, with significant differences by department found for respondents’ confidence in their awareness of trafficking worldwide, ability to provide trauma-informed care, understanding of the confidentiality issues involved, and ability to ensure patient’s safety when the patient is a trafficked person. Whether there should be an ICD code for suspected or confirmed trafficking and whether they had had relevant training within the last three years also differed by department (S5 Table). Tenure, or the number of years as a healthcare professional, influenced awareness of where HT occurs, ability to provide culturally-sensitive care, understanding of the role of healthcare professionals in the prevention of HT, and agreement that continuity of care is an acute problem for trafficked persons (S6 Table).

To investigate whether healthcare encounters with patients who are suspected or known to be trafficked persons is a geographically concentrated problem (e.g., in more urban areas, in close proximity to major highways, or in areas containing a hospital) or geographically dispersed throughout the healthcare system, responses were examined by three-digit workplace zip codes. Reported encounters with trafficked patients varied by three-digit zip codes (p = 0.0364), and responses reported in the Pennsylvania state capitol region (170**) were statistically different from others (p = 0.0114). Encounters with suspected or known HT patients did not vary among zip codes with hospitals and those without (S7 Table).

Each item assessing ability, understanding, and preparedness varied by the respondents’ confidence in their ability to define HT (p-values ranging from <0.0001 to 0.0025) (S8 Table). Level of agreement with the items assessing HT as a perceived problem in the geographic area; encounters with patients suspected or known to be a trafficked person; and recent relevant training varied by ability to define HT as well (p = 0.0006, p = 0.0051, and p = 0.0002, respectively).

Responses to all items assessing ability, understanding, and preparedness varied by the respondents’ agreement with the statement “While working at my current institution, I have encountered a patient whom I suspected or knew was a trafficked person” (p-values ranging from <0.0001 to 0.0426). Levels of agreement with the items regarding the adequacy of the institution’s training and attendance at a recent relevant training also varied by response to the encounter statement (each at p<0.0001) (S9 Table).

Several other analyses were performed that did not uncover anything of significance. For example, the ability to define HT did not vary by age, and perspectives regarding use of an ICD code, biometric tools, and DNA identifiers did not vary by educational attainment. A statistically significant difference was detected in the level of agreement with “Referrals to non-medical services (such as housing, employment, immigration, food, or legal services) are not a healthcare professional’s responsibility” based on role as a healthcare professional, race/ethnicity, and gender identity. Interpretation of this finding requires caution, as it could be spurious and attributable to confusion from double-negative phrasing.

## Discussion

Our initial data pull on EHR for records on potential cases of HT was unsuccessful. Since our study, HT-specific ICD-10 codes have been released, but the lack of data on trafficking cases in the Geisinger health system indicated a need for a broader investigation into how cases of trafficking are documented. This is what prompted our mixed-methods research involving (1) key informant interviews, and (2) subsequent survey of physicians and registered nurses to examine how HT is recognized in healthcare systems and subsequently communicated through patient care. A major impetus for this study was to explore perspectives regarding the potential application of tools developing as part of the precision medicine movement (namely, biometrics such as genetic data and EHR data-sharing infrastructure) to improve the care of trafficked patients and begin to consider the foreseeable ethical, legal, and social issues (including but not limited to privacy, contextual integrity, and management of risks associated with unintended secondary data access and use) that would need to be addressed in order for such an application to be designed and implemented responsibly. Such studies remain difficult to pursue because of the continued under-recognition of HT as a local problem and persistent biases about HT in healthcare settings. Implicit and explicit bias continue in healthcare settings, limiting the identification of (and subsequent assistance provided to) trafficked persons. Leveraging precision medicine tools to improve care of trafficked patients will remain ineffective if their development does not coincide with a broader effort to improve acknowledgment of HT as a serious public health matter.

Despite the limitations of our study (see S4 Appendix), our interviews with healthcare providers at large, well-funded, and well-networked hospitals provided insight into the dearth of resources and lack of a cohesive approach to managing healthcare for trafficked persons. The key informant interviews also provided us with a better appreciation of the knowledge deficits hindering precision medicine advocates from ensuring a design with these vulnerable patient populations in mind. Interviews highlighted a strong desire among providers to engage with the community and learn how best to support patients in endangered environments, and informants cited opportunities to educate providers on what HT is, what resources are available for providers, and how best to manage a suspected HT patient. Likening care of trafficked persons to domestic violence victims was a pertinent message and entirely translatable in practice. Further data on where gaps in education might exist would be useful in formulating curricula and resources for providers. Monitoring the use of the new HT-specific ICD-10 codes and examining healthcare providers’ experiences with those codes will be important areas of inquiry for future research.

We stress the importance of pondering (and empirically studying) how biometrics might improve the health and well-being of trafficked persons. When connected to a patient’s EHR, biometrics might improve continuity of care. This improvement offered by a consistent identifier (such as a DNA fingerprint) could be substantial for trafficked patients who, despite requiring substantial and diverse healthcare, have no longitudinal EHR. Trafficked persons commonly use false names and identification to access the healthcare system (1) to protect themselves and their families from retribution by the people exploiting them and (2) out of fear of law enforcement [34]. Trafficked persons also require substantial healthcare depending on the context of their exploitation. For instance, a minor female sex worker might require contraceptives, obstetric care, STD clinic visits, or ER treatment following violent incidents. For each visit, she might use a different identity to access care. With biometrics her care could be streamlined. Using genetic information in combination with a trafficked persons database could also enable identification of missing children and reunification of trafficked minors with their parents [34].

Biometrics are already under consideration by healthcare systems, outside the context of detecting trafficked persons, for reducing patient misidentification and medical error [47–48]. While no studies to date have evaluated the rates of patient misidentification before and after implementation of biometrics in a healthcare system, palm and retinal scanning technologies are being implemented to eliminate error at the bedside and to differentiate patients with similar names and other identifiers. Moreover, as genomic sequencing is routinized in care (as has already been initiated at Geisinger), it seems appropriate to consider the many uses and potential returns on that institutional investment. At the same time, use of biometric data intended to improve health care could introduce unexpected harms without proper protections in place and transparency of how data is collected, used, shared, and stored. As our interviews illuminated, some hesitation surrounding biometrics stems from an incomplete understanding of how such information would be gathered and communicated throughout various healthcare systems. As modern data protection and data sharing models develop (necessities for the advancement of precision medicine), understanding key aspects of how data are shared is paramount to gaining trust of patients with their biometric information. Most importantly, an evaluation of the potential risks of harm from biometrics (including privacy and contextual integrity) along with the potential benefits is needed. We must understand how the data are handled, how harms from misuse are mitigated, and how potential secondary non-medical purposes for the data are both anticipated and communicated. Clearing misconceptions on how biometrics are (and can be) used should be a priority.

Detecting trafficked persons and recognizing HT patterns require a sustained documentation effort. This documentation is important for patient care, prosecution of trafficking cases, and ongoing research (e.g., of health outcomes or effectiveness of medical and policy interventions). What to document in EHRs and how that documentation is used–in a clinic, within one healthcare system, with other healthcare systems, and outside healthcare–are ripe matters for research and policy development. Our findings are consistent with the growing public policy perception that trafficked persons are accessing healthcare but that their trafficked status is going largely unnoticed. Additional empirical data from other systems and locales are needed to understand the issues more fully. The recent establishment of ICD-10 codes could improve EHR documentation, assuming healthcare providers are properly trained to use them consistently. The new ICD-10 codes and the application of precision medicine principles—including broad data sharing across healthcare systems and integration of biometric data (such as genetic information)—could bring to light the scope of healthcare utilization by trafficked persons and the extent of their unmet needs. For unidentified patients and patients whose identity is actively concealed (by themselves or by traffickers), there are no longitudinal EHRs or consistent identifiers. Biometrics could lighten the digital burden and enable patient-centered, multifaceted care to address the needs of this patient population. The potential for expanded biometrics and data sharing to bring vulnerable patients out of the EHR shadows and improve their care and well-being deserves serious attention and policy consideration.

While our research efforts did not enable us to gain deep understanding about the potential for biometric identifiers (including genetic information) and the unique opportunities and challenges that precision medicine efforts might pose for trafficked patients, our survey of healthcare professionals is the first to examine this approach to continuity of care and has distinct scientific merit worth reporting. Notably, this survey involved physicians and registered nurses employed at Geisinger, a health care system recognized among the leaders of genomic and precision medicine efforts in the United States. As a result, the findings might be relevant starting points for any institution concerned about the possibility of its precision medicine efforts overlooking this vulnerable patient population or having unintended impacts, and the survey instrument (S2 Instrument) could provide a consistent way to evaluate the physicians and nurses throughout the United States.

Our survey findings confirm that healthcare providers—physicians and nurses alike—generally lack the necessary ability, understanding, and preparedness to provide patient-centered care for patients who are trafficked persons but that these providers are nevertheless eager to learn. Similar findings have recently been reported [49]. While the number of providers who indicated that they have encountered a patient known or suspected to be a trafficked person is relatively low, the vast majority of physicians and registered nurses lack confidence in their ability to spot the warning signs or indicators that a patient is a trafficked person. It is likely that those enduring modern-day slavery in our midst are going largely unnoticed. This lack of confidence in caring for trafficked patients found in our survey is consistent with findings from a similar survey recently reported [49]. Our survey findings suggest that policy and technical interventions to improve care for trafficked patients might have sufficient support for pilot testing (e.g., biometric tools for identification that could improve patient safety and continuity of care; See Table 4, Items 28–29). While a review of the literature might suggest that healthcare professionals in more urban areas are likely to be better equipped to handle the challenges of healthcare delivery for trafficked persons, our survey did not reveal variation based among rural and non-rural residences. Our examination of work place zip codes suggests that even though HT is a geographically distributed problem across this healthcare system, efforts to educate providers should not be limited to major hospital locations. Moreover, while there appeared to be a concentration of HT patient encounters in the Pennsylvania capitol region, this finding likely reflects heightened awareness from anti-HT efforts in that area rather than increased incidence. For example, Sinha, Tashakor, and Pinto reported recently that those who have had some educational training to identify trafficked patients show increased levels of knowledge, comfort, and confidence in caring for trafficked patients [49].

Meaningful efforts to design and implement precision medicine initiatives in a way that is inclusive and optimizes its impacts are unlikely to succeed without concurrent efforts to increase general awareness of and preparedness to care for trafficked persons. Much of the biomedical field remains ill-equipped to engage in the deliberative discourse necessary to ensure that everyone enjoys the benefits and risks of precision medicine. We call upon others to join us in relevant research in this area, prepared with these preliminary data and the newly released ICD codes.

## Supporting information

S1 Appendix Human trafficking definitions. (DOCX)

S2 Appendix Geisinger as an ideal study site. (DOCX)

S3 Appendix Study team. (DOCX)

S4 Appendix Study limitations. (DOCX)

S1 Instrument Interview guide with piloted survey instrument. (PDF)

S2 Instrument Validated survey instrument. (PDF)

S1 Table Survey responses by physician or registered nurse status. (DOCX)

S2 Table Survey responses by rural or non-rural (suburban or urban) residence. (DOCX)

S3 Table Survey responses by race/ethnicity. (DOCX)

S4 Table Survey responses by gender identity. (DOCX)

S5 Table Survey responses by department. (DOCX)

S6 Table Survey responses by years in the healthcare profession. (DOCX)

S7 Table Survey responses to “while working at my current institution, I have encountered a patient whom I suspected or knew was a trafficked person” by 3-Digit Work Zip Code. (DOCX)

S8 Table Survey responses by confidence in “I can define human trafficking”. (DOCX)

S9 Table Survey response by agreement with “while working at my current institution, I have encountered a patient whom I suspected or knew was a trafficked person”. (DOCX)

S1 Data Interview data. (XLSX)

S2 Data Survey data. (XLSX)
