# Supplementary material for: Caring for trafficked and unidentified patients in the EHR shadows: Shining a light by sharing the data
Source: PLoS One. 2019 Mar 14;14(3):e0213766. doi: 10.1371/journal.pone.0213766 (PMC6417704; doi:10.1371/journal.pone.0213766)
Supplement: S1 Appendix — (DOCX) [file pone.0213766.s001.docx]

**S1 Appendix: Human Trafficking Definitions**

*Article 3 of the “Palermo Protocol”* [18] – “The recruitment, transportation, transfer, harbouring or receipt of persons, by means of the threat or use of force or other forms of coercion, of abduction, of fraud, of deception, of the abuse of power or of a position of vulnerability or of the giving or receiving of payments or benefits to achieve the consent of a person having control over another person, for the purposes of exploitation. Exploitation shall include, at a minimum, the exploitation of the prostitution of others or other forms of sexual exploitation, forced labour or services, slavery or practices similar to slavery, servitude or the removal of organs.”

*Victims of Trafficking and Violence Protection Act of 2000 (TVPA)* [19] – (1) “sex trafficking in which a commercial sex act is induced by force, fraud, or coercion, or in which the person induced to perform such act has not attained 18 years of age,” and (2) “the recruitment, harboring, transportation, provision, or obtaining of a person for labor or services, through the use of force, fraud, or coercion for the purpose of subjection to involuntary servitude, peonage, debt bondage, or slavery.”
