# Supplementary material for: Caring for trafficked and unidentified patients in the EHR shadows: Shining a light by sharing the data
Source: PLoS One. 2019 Mar 14;14(3):e0213766. doi: 10.1371/journal.pone.0213766 (PMC6417704; doi:10.1371/journal.pone.0213766)
Supplement: S2 Appendix — (DOCX) [file pone.0213766.s002.docx]

**S2 Appendix: Geisinger as an ideal study site for a preparatory-to-research EHR data pull.**

Geisinger adopted the Epic® Corporation (Verona, WI) EHR in 1996, incorporated a site using the Cerner Corporation (North Kansas City, MO) EHR, and fully implemented and integrated the EHR across all its ambulatory and inpatient sites of care. It contains health records for more than 3 million unique patients. Geisinger adopted a comprehensive system-wide data warehouse in 2006. Members of the Geisinger Phenomic Analytics & Clinical Data Core serve as institutional data brokers serve as an important firewall between clinical and research activities to enable research without compromising patient confidentiality. Geisinger serves approximately 4.2 million residents in 45 counties in Pennsylvania and six counties in New Jersey and involves an integrated network of 13 hospitals as well as 100 primary, 167 specialty, and 25 urgent care clinics. In addition to its reputation for health care innovation, Geisinger is known as a leader in precision medicine with its MyCode® Community Health Initiative, which has more than 200,000 patient-participants and has returned actionable genomic results to more than 1,000 patient-participants as of November 1, 2018.
