# Supplementary material for: Caring for trafficked and unidentified patients in the EHR shadows: Shining a light by sharing the data
Source: PLoS One. 2019 Mar 14;14(3):e0213766. doi: 10.1371/journal.pone.0213766 (PMC6417704; doi:10.1371/journal.pone.0213766)
Supplement: S3 Appendix — (DOCX) [file pone.0213766.s003.docx]

**S3 Appendix: Study Team**

For COREQ reporting purposes we include the following additional information about our study team and approach to the interviews and qualitative research:

1. This study was led by JKW. JKW has a JD and PhD and is experienced with social science research (including qualitative and quantitative methods) regarding vulnerable populations, including specifically trafficked persons.

2. All interviews were conducted by JKW and SK, experienced scholars in ELSI research (ethical, legal, and social implications of genomics). SK has a MS and is also experienced with with social science research (including qualitative and quantitative methods) regarding vulnerable populations, including specifically trafficked persons. JKW and SK developed the interview guide collaboratively.

3. The interviewers and interviewees did not have a prior relationship. Health care providers at Duke study site were randomly selected from a list of physicians. Snowball sampling was used to select stakeholder informants at Geisinger. An informational sheet was provided with the initial contact to establish a relationship between the interviewers and interviewees and communicate the goals and purposes of the study. Interviewers also described during the interview that their professional interest was focused on potential applications of biometrics and genetic information. Interviews typically lasted one hour in length, and interviewers conducted new interviews until data saturation was reached. No repeat interviews were conducted for this study.

5. Interviews were recorded and transcribed. EH, a research intern with proper human subjects research training and under the supervision of JKW, created the transcriptions. A review of the relevant scholarly literature and other research support was provided by undergraduate research interns LW (under supervision of JKW) and VG (under supervision of SK).

6. SK and EH independently coded the transcripts independently to identify emerging themes and corresponding quotes. The two coders met to discuss their independent findings and determined them to be consistent. Eight major themes were identified and illustrative quotations were agreed upon by the coders. No generalizable minor themes were developed for this study given the small size and limited topical coverage.

7. SL has a PhD and is an expert in population health research and survey methodology. She provided guidance on the recruitment approach and design of the survey.

8. AY is an expert in statistics and employed with the statistics core at Geisinger. She performed all statistical analyses.
