# Supplementary material for: Caring for trafficked and unidentified patients in the EHR shadows: Shining a light by sharing the data
Source: PLoS One. 2019 Mar 14;14(3):e0213766. doi: 10.1371/journal.pone.0213766 (PMC6417704; doi:10.1371/journal.pone.0213766)
Supplement: S4 Appendix — (DOCX) [file pone.0213766.s004.docx]

**S4 Appendix: Limitations**

**A. Limitations of EHR Data Pull**

The EHR data pull underscored the difficulty in studying HT as a public health problem, as our data request was both over- and under-inclusive and too non-specific to generate useful information. Our data pull method could be improved with better understanding from this and subsequent studies. For example, we now understand the “Trauma 181” code might refer to one of many recycled numbers, so a data request for a range (1-200) might yield results from historical data. The availability of new ICD-10 codes specifically for HT might yield results from future cases as well. The potential data pull could be further improved with closer consultations with anti-trafficking professionals who could provide insights regarding signs of HT discernable from the EHR (e.g., signs of injections or needle use). It is important to recall that our study was not hypothesis-testing and did not involve chart reviews to confirm actual cases of suspected HT.

**B. Limitations of Key Informant Interviews**

We were unable to access all types of providers for our interviews or access the full range of hospital staff who might interact with a trafficked person. While diversity in informant recruitment was a priority, we were unable to interview social workers or ER doctors in North Carolina or OB/GYN providers in Pennsylvania, and interviewees lacked age and other diversity desirable in a larger study.

**C. Limitations of Survey**

While our survey was system-wide at a large, integrated health system in Pennsylvania, it is possible that important healthcare professionals were not in the sampling frame (e.g., front desk personnel, security officers, executive leadership, etc.) but would have important perspectives and roles in detecting and stopping HT and also in making institutional decisions about resources and protocols regarding the delivery of care for trafficked persons.

**D. Overall Limitations of this Mixed-Methods Approach**

While we do not shy away from discussing the motivating interest for our study and stress our view that biometrics must be examined (particularly as use of genomic information as routine care and large-scale precision medicine initiatives are now underway), we recognize that the use of biometrics is not a panacea or without potential harms. Privacy, data security, and the varying perspectives regarding appropriate secondary uses of the data (for medical and non-medical purposes) are all worthy of study.
