# Supplementary material for: Caring for trafficked and unidentified patients in the EHR shadows: Shining a light by sharing the data
Source: PLoS One. 2019 Mar 14;14(3):e0213766. doi: 10.1371/journal.pone.0213766 (PMC6417704; doi:10.1371/journal.pone.0213766)
Supplement: S1 Instrument — (PDF) [file pone.0213766.s005.pdf]

KEY INFORMANT INTERVIEW GUIDE

[Instructions for Interviewer: After beginning the audio recording, start interview recording by stating the date, the time, the names of the interviewer(s) and interviewee, and asking the interviewee "Do you give us your consent to participate in this research?" Proceed to ask about the basic information about the interviewee that we would otherwise get from the sociodemographic survey we plan to administer later in a separate study.]

1. **I am going to ask you some questions about the health care of human trafficking victims and unidentified persons. How would you describe what that phrase 'human trafficking' means? How have you previously heard about human trafficking?**

2. **Where do you think victims of human trafficking are from?**

3. **Does anyone at your institution focus on health care access for human trafficking victims?**

4. **Is human trafficking a problem in your geographic area? In the population that you serve in your health care practice?**
    a. How would you describe the <u>magnitude</u> of health care services needed by human trafficking victims in your geographic area? In the population that you serve?
    b. How would you describe <u>the type</u> of health care services needed by human trafficking victims in your geographic area? In the population that you serve?

5. **How do human trafficking victims access health care at your institution?**
    a. What types of health professionals encounter human trafficking victims in your institution? (e.g., nurses, PAs, physicians)
    b. Which health system departments or specialty areas encounter human trafficking victims as patients?

6. **Can you describe the policies at your institution for managing health care of unidentified patients?**

7. **Does your institution have any policies related to health care delivery for human trafficking victims?**
    a. How does your institution define human trafficking?
    b. How would medical staff recognize human trafficking?
    c. How are unidentified patients managed? How are they screened for human trafficking?
    d. How does your institution manage or screen for provision of false identities by patients and/or human trafficking victims?
    e. Are there special services or care lines available for patients who are human trafficking victims?
    f. Is there a training policy at your institution so employees know how to identify and help a human trafficking victim?

8. **Are there particular EHR codes to note potential human trafficking victims? Unidentied persons? False identitites?**

9. **What would you do if you recognize or suspect that a patient is a human trafficking victim?**
    a. Whom would you notify within the health care system? (e.g., social worker, supervisors)
    b. Whom would you notify outside of the health care system? (e.g., social services, law enforcement)
    c. What special steps might you take to assist a victim?

10. **What would help you or your institution be better able to respond to health care needs of human trafficking victims?**

11. **Would use of some form of biometrics help continuity of health care of patients that might provide false identification or have no identification? What might you suggest? (e.g., fingerprints, DNA)**

12. **Is there anything else we haven't covered during this interview that you think we should know about if we want to learn more about caring for trafficked persons?**

13. **Would you be willing to complete a pilot survey for us? [If yes, hand them the survey or, if the survey is by phone, coordinate how the interviewee will receive and deliver the responses via email.]**

[Interviewer: before ending the interview, remember to administer the sociodemographic questionnaire]

Pilot Survey **Note: Revisions will be made to this draft following key informant interviews to maximize appropriateness of survey questions to address research questions of interest. Survey may be administered on paper or electronically.*

For each item, select the confidence level that best reflects your own abilities, understanding, or preparedness.

Not Confident	Hesitant	Confident	Very Confident
**1**	**2**	**3**	**4**

1. I can define "human trafficking."	1	2	3	4
2. I can identify multiple types of human trafficking.	1	2	3	4
3. I know how human trafficking occurs.	1	2	3	4
4. I know where human trafficking occurs.	1	2	3	4
5. I am aware of the extent of human trafficking occurring in my state.	1	2	3	4
6. I am aware of the extent of human trafficking occurring worldwide.	1	2	3	4
7. I understand the physical health consequences of human trafficking.	1	2	3	4
8. I understand the psychological health consequences of human trafficking.	1	2	3	4
9. I know the warning signs or indicators that a patient is a trafficked person.	1	2	3	4
10. I know how to communicate effectively with a patient suspected of being a trafficked person.	1	2	3	4
11. I know how to provide trauma-informed medical care for a patient suspected of being a trafficked person.	1	2	3	4
12. I know how to provide culturally-sensitive medical care for a patient suspected of being a trafficked person.	1	2	3	4
13. I know where trafficked persons can obtain housing assistance and how to refer patients to those resources.	1	2	3	4
14. I know where trafficked persons can obtain legal assistance and how to refer patients to those resources.	1	2	3	4
15. I know where trafficked persons can obtain immigration assistance and how to refer patients to those resources.	1	2	3	4
16. I know where trafficked persons can obtain employment assistance and how to refer patients to those resources.	1	2	3	4
17. I know where trafficked persons can obtain food assistance and how to refer patients to those resources.	1	2	3	4

	Not Confident	Hesitant	Confident	Very Confident
	1	2	3	4
18. I understand the <u>medical record documentation issues</u> related to caring for a patient suspected of being a trafficked person.	1	2	3	4
19. I understand the <u>confidentiality issues</u> related to caring for a patient suspected of being a trafficked person.	1	2	3	4
20. I understand the <u>reporting issues</u> related to caring for a patient suspected of being a trafficked person.	1	2	3	4
21. I know how to ensure <u>my own</u> security and safety as a health care provider of a trafficked person.	1	2	3	4
22. I know how to ensure <u>my patient's</u> security and safety when I suspect or know the patient is a trafficked person.	1	2	3	4
23. I understand the role of health care professionals in the prevention of human trafficking.	1	2	3	4

24. Which of the following do you consider to be examples of human trafficking [RANDOMIZE, SELECT ALL THAT APPLY]
    a. Consensual sex work
    b. Sex work through a third party (such as a pimp)
    c. Debt bondage for smuggling a person across borders for exploitation
    d. Payment for smuggling a person across borders
    e. Selling a child for adoption
    f. Paying a woman to give up her child for adoption
    g. Selling of a person's own organs or body parts
    h. Selling another person's organs or body parts
    i. Selling a person's own reproductive samples (eggs, sperm) across borders
    j. Forced collection of a person's biological sample for government use
    k. Forced collection of a person's biological sample for profit
    l. Forced or bonded labor
    m. Child labor
    n. Child pornography
    o. Child prostitution
    p. Forced use of a child for military
    q. Forced marriage of a child
    r. Forced marriage of an adult

Please indicate your level of agreement with the following statements.

	Strongly Disagree	Disagree	Agree	Strongly Agree
	**1**	**2**	**3**	**4**
25. There should be a specific ICD code for use when a patient is suspected or confirmed as a trafficked person.	1	2	3	4
26. Continuity of care is more important for trafficked persons, than it is for standard patients.	1	2	3	4
27. The use of DNA identifiers (or other biomarkers) would improve the continuity of care for trafficked persons.	1	2	3	4
28. Human trafficking is not a problem in the geographic area where I work as a health care professional.	1	2	3	4
29. Referrals to non-medical services (such as housing, employment, immigration, food, or legal services) are not a health care professional's responsibility.	1	2	3	4
30. Geisinger has trained adequately its health care providers to care for patients who are trafficked persons.	1	2	3	4
31. While working at Geisinger, I have encountered a patient whom I suspect or know was a trafficked person.	1	2	3	4
32. Within the last three years, I have attended training (such as an in-person or online course) related to human trafficking and health care.	1	2	3	4
33. I want to learn more about identification, intervention, and prevention of human trafficking.	1	2	3	4

1. Which of the following best describes your role as a health care professional?
   a. Nurse
   b. Physician
   c. Other ___________________________
   d. Prefer not to answer

2. In which department do you primarily work?
   a. Emergency
   b. OB/GYN
   c. Other ___________________________
   d. Prefer not to answer

3. At which Geisinger location do you primarily work?
   a. Geisinger Medical Center
   b. Geisinger Community Medical Center
   c. Geisinger Wyoming Valley Medical Center
   d. Janet Weis Children's Hospital
   e. Geisinger Bloomsburg Hospital
   f. Geisinger Lewistown Hospital
   g. Geisinger Shamokin Area Community Hospital
   h. Geisinger South Wilkes-Barre
   i. Geisinger Holy Spirit
   j. AtlantiCare Health System
   k. Marworth Alcohol and Chemical Dependency Treatment Center
   l. Other _____________________________
   m. Prefer not to answer

4. How many years have you worked as a health care professional?
   a. Fewer than 10 years
   b. 10-19 years
   c. 20-29 years
   d. 30 years or more
   e. Prefer not to answer

5. In which age group do you belong?
   a. 18 to 25 years old
   b. 26 to 35 years old
   c. 36 to 45 years old
   d. 46 to 55 years old
   e. 56 to 65 years old
   f. 66 to 75 years old
   g. 76 years and older
   h. Prefer not to answer

6. What is the highest grade or year of school you completed?
   a. Less than Grade 12 (did not graduate high school)
   b. Grade 12 or GED (High school graduate)
   c. 1 to 3 years after high school (Some college, Associate's degree, or technical school)
   d. College 4 years or more (College graduate)
   e. Advanced degree (Master's, Doctorate, etc.)
   f. Prefer not to answer

7. Which of the following best describes the area in which you live?
   a. Rural
   b. Suburban
   c. Urban
   d. Prefer not to answer

8. Were you born in the USA?
   a. Yes
   b. No (Provide the name of the country in which you were born: __________________________)
   c. Prefer not to answer

9. Which categories describe you? Select all that apply. Note, you may select more than one group.
   a. American Indian or Alaska Native
   b. Asian
   c. Black, African American, or African
   d. Hispanic, Latino, or Spanish
   e. Middle Eastern or North African
   f. Native Hawaiian or other Pacific Islander
   g. White, European American, or European
   h. None of these fully describe me (optional free text answer)
   i. Prefer not to answer

10. What was your biological sex assigned at birth?
    a. Female
    b. Male
    c. Intersex
    d. Prefer not to answer

11. What term best expresses how you describe your gender identity?
    a. Man
    b. Woman
    c. Non-binary
    d. Transgender
    e. None of these describe me
    f. Prefer not to answer

12. Which term best represents how you think of your sexual orientation?
    a. Gay
    b. Lesbian
    c. Straight (that is, not gay or lesbian)
    d. Bisexual
    e. None of these describe me
    f. Prefer not to answer
