# Supplementary material for: Caring for trafficked and unidentified patients in the EHR shadows: Shining a light by sharing the data
Source: PLoS One. 2019 Mar 14;14(3):e0213766. doi: 10.1371/journal.pone.0213766 (PMC6417704; doi:10.1371/journal.pone.0213766)
Supplement: S2 Instrument — (PDF) [file pone.0213766.s006.pdf]

Caring for Trafficked Persons and Unidentified Patients

1. For each item, select the confidence level that best reflects your own abilities, understanding, or preparedness.

	Not Confident	Hesitant	Confident	Very Confident
I can define “human trafficking.”	○	○	○	○
I can identify multiple types of human trafficking.	○	○	○	○
I know where human trafficking occurs.	○	○	○	○
I am aware of the extent of human trafficking occurring in my state.	○	○	○	○
I am aware of the extent of human trafficking occurring worldwide.	○	○	○	○
I understand the physical health consequences of human trafficking.	○	○	○	○
I understand the psychological health consequences of human trafficking.	○	○	○	○
I know the warning signs or indicators that a patient is a trafficked person.	○	○	○	○
I know how to communicate effectively with a patient suspected of being a trafficked person.	○	○	○	○
I know how to provide trauma-informed medical care for a patient suspected of being a trafficked person.	○	○	○	○
I know how to provide culturally-sensitive medical care for a patient suspected of being a trafficked person.	○	○	○	○
I know where trafficked persons can obtain housing assistance.	○	○	○	○
I know where trafficked persons can obtain legal assistance.	○	○	○	○
I know where trafficked persons can obtain immigration assistance.	○	○	○	○
I know where trafficked persons can obtain employment assistance.	○	○	○	○
I know where trafficked persons can obtain food assistance.	○	○	○	○
I know how to refer trafficked persons to non-medical services (such as housing, legal, immigration, employment, and food assistance resources).	○	○	○	○
I understand the medical record documentation issues related to caring for a patient suspected of being a trafficked person.	○	○	○	○
I understand the confidentiality issues related to caring for a patient suspected of being a trafficked person.	○	○	○	○
I understand the law enforcement reporting issues related to caring for a patient suspected of being a trafficked person.	○	○	○	○
I know how to ensure my own security and safety as a healthcare provider of a trafficked person.	○	○	○	○

	Not Confident	Hesitant	Confident	Very Confident
I know how to ensure my patient’s security and safety when I suspect or know the patient is a trafficked person.	◯	◯	◯	◯
I understand the role of healthcare professionals in the prevention of human trafficking.	◯	◯	◯	◯

2. Please indicate your level of agreement with the following statements.

	Strongly Disagree	Disagree	Agree	Strongly Agree
Referrals to non-medical services (such as housing, employment, immigration, food, or legal services) are not a healthcare professional’s responsibility.	○	○	○	○
Human trafficking is not a problem in the geographic area where I work as a healthcare professional.	○	○	○	○
Continuity of care is an acute problem for trafficked persons.	○	○	○	○
There should be a specific ICD code for use when a patient is suspected or confirmed as a trafficked person.	○	○	○	○
The use of biometric tools (like palm readers, fingerprinting, and retinal or iris scans) would improve patient safety.	○	○	○	○
The use of DNA identifiers (or other biomarkers) would improve the continuity of care for trafficked persons.	○	○	○	○
My current institution has trained adequately its healthcare providers to care for patients who are trafficked persons.	○	○	○	○
While working at my current institution, I have encountered a patient whom I suspected or knew was a trafficked person.	○	○	○	○
Within the last three years, I have attended training (such as an in-person or online course) related to human trafficking and healthcare.	○	○	○	○
I want to learn more about identification, intervention, and prevention of human trafficking.	○	○	○	○

Caring for Trafficked Persons and Unidentified Patients

3. Which of the following best describes your role as a health care professional?

- ○ Physician
- ○ Nurse
- ○ Prefer not to answer
- ○ Other (please specify)

4. In which department do you primarily work?

- ○ Emergency
- ○ OB/GYN
- ○ Pediatrics
- ○ Psychiatry
- ○ Prefer not to answer.
- ○ Other (please specify)

5. What is the zip code for the place where you primarily work as a health care professional?

6. How many years have you worked as a health care professional?

- ○ Fewer than 10 years
- ○ 10-19 years
- ○ 20-29 years
- ○ 30 years or more
- ○ Prefer not to answer.

7. In which age group do you belong?

- ◯ 18 to 25 years old
- ◯ 26 to 35 years old
- ◯ 36 to 45 years old
- ◯ 46 to 55 years old
- ◯ 56 to 65 years old
- ◯ 66 to 75 years old
- ◯ 76 years and older
- ◯ Prefer not to answer.

8. What is the highest grade or year of school you completed?

- ◯ Less than Grade 12 (did not graduate high school)
- ◯ Grade 12 or GED (high school graduate)
- ◯ 1 to 3 years after high school (some college, Associate's degree, or technical school)
- ◯ College 4 years or more (college graduate)
- ◯ Advanced degree (Master's, Doctorate, etc.)
- ◯ Prefer not to answer.

9. Which of the following best describes the area in which you live?

- ◯ Rural
- ◯ Suburban
- ◯ Urban
- ◯ Prefer not to answer.

10. Were you born in the USA?

- ◯ Yes
- ◯ No
- ◯ Prefer not to answer

11. Which categories describe you? Select all that apply. Note, you may select more than one group.

- ◯ American Indian or Alaska Native
- ◯ Asian
- ◯ Black, African American, or African
- ◯ Hispanic, Latino, or Spanish
- ◯ Middle Eastern or North African
- ◯ Native Hawaiian or other Pacific Islander
- ◯ White, European American, or European
- ◯ Prefer not to answer.
- ◯ None of these fully describe me. (please specify)

12. What term best expresses how you describe your gender identity?

- ◯ Man
- ◯ Woman
- ◯ Non-Binary
- ◯ Transgender
- ◯ Prefer not to answer.
- ◯ None of these describe me. (please specify)

Caring for Trafficked Persons and Unidentified Patients

You have finished the survey. Thank you for your participation in this survey. Please close your internet browser now.

Note: If you do not close your browser at the end of this survey, it is possible that SurveyMonkey will show you advertising or ask you to complete other surveys that are not related to this study. Please close your browser now to avoid any confusion.
