# Supplementary material for: Caring for trafficked and unidentified patients in the EHR shadows: Shining a light by sharing the data
Source: PLoS One. 2019 Mar 14;14(3):e0213766. doi: 10.1371/journal.pone.0213766 (PMC6417704; doi:10.1371/journal.pone.0213766)
Supplement: S1 Table — (DOCX) [file pone.0213766.s007.docx]

**S1 Table. Survey Responses by Physician or Registered Nurse Status**

|  | **Physician**  **N=162** | **Nurse**  **N=738** | **Fisher’s Exact Test p-value** |
| --- | --- | --- | --- |
| **Confident of ability, understanding and preparedness, N (%)** |  |  |  |
| I can define “human trafficking.” | 105 (64.8) | 524 (71) | 0.1304 |
| I can identify multiple types of human trafficking. | 73 (45.1) | 281 (38.1) | 0.1099 |
| I know where human trafficking occurs. | 39 (24.1) | 196 (26.8) | 0.5540 |
| I am aware of the extent of human trafficking occurring in my state. | 22 (13.8) | 112 (15.2) | 0.7143 |
| I am aware of the extent of human trafficking occurring worldwide. | 55 (34.2) | 258 (35.1) | 0.8555 |
| I understand the physical health consequences of human trafficking. | 90 (55.6) | 410 (55.8) | 1.0000 |
| I understand the psychological health consequences of human trafficking. | 93 (57.8) | 442 (60.3) | 0.5944 |
| I know the warning signs or indicators that a patient is a trafficked person. | 23 (14.3) | 89 (12.1) | 0.4324 |
| I know how to communicate effectively with a patient suspected of being a trafficked person. | 18 (11.1) | 62 (8.5) | 0.2878 |
| I know how to provide trauma-informed medical care for a patient suspected of being a trafficked person. | 23 (14.2) | 96 (13) | 0.7014 |
| I know how to provide culturally-sensitive medical care for a patient suspected of being a trafficked person. | 27 (16.7) | 154 (20.9) | 0.2361 |
| I know where trafficked persons can obtain housing assistance. | 15 (9.3) | 53 (7.2) | 0.4113 |
| I know where trafficked persons can obtain legal assistance. | 11 (6.8) | 49 (6.7) | 1.0000 |
| I know where trafficked persons can obtain immigration assistance. | 9 (5.6) | 24 (3.3) | 0.1671 |
| I know where trafficked persons can obtain employment assistance. | 8 (4.9) | 39 (5.3) | 1.0000 |
| I know where trafficked persons can obtain food assistance. | 18 (11.1) | 89 (12.1) | 0.7899 |
| I know how to refer trafficked persons to non-medical services (such as housing, legal, immigration, employment, and food assistance resources). | 18 (11.3) | 69 (9.5) | 0.4662 |
| I understand the medical record documentation issues related to caring for a patient suspected of being a trafficked person. | 18 (11.1) | 48 (6.5) | 0.0654 |
| **I understand the confidentiality issues related to caring for a patient suspected of being a trafficked person.** | **47 (29)** | **308 (42)** | **0.0025** |
| I understand the law enforcement reporting issues related to caring for a patient suspected of being a trafficked person. | 24 (14.9) | 115 (15.6) | 0.9045 |
| I know how to ensure my own security and safety as a healthcare provider of a trafficked person. | 25 (15.4) | 126 (17.3) | 0.6437 |
| I know how to ensure my patient’s security and safety when I suspect or know the patient is a trafficked person. | 22 (13.6) | 143 (19.5) | 0.0926 |
| I understand the role of healthcare professionals in the prevention of human trafficking. | 29 (18) | 162 (22.1) | 0.2885 |
| **Agree with the following statements, N (%)** |  |  |  |
| Referrals to non-medical services (such as housing, employment, immigration, food, or legal services) are not a healthcare professional’s responsibility. | 35 (21.6) | 67 (9.1) | **<0.0001** |
| Human trafficking is not a problem in the geographic area where I work as a healthcare professional. | 42 (26.1) | 142 (19.4) | 0.0670 |
| Continuity of care is an acute problem for trafficked persons. | 155 (95.7) | 648 (88.9) | **0.0082** |
| There should be a specific ICD code for use when a patient is suspected or confirmed as a trafficked person. | 124 (77.5) | 565 (78.7) | 0.7501 |
| The use of biometric tools (like palm readers, fingerprinting, and retinal or iris scans) would improve patient safety. | 117 (72.7) | 518 (71.9) | 0.9227 |
| The use of DNA identifiers (or other biomarkers) would improve the continuity of care for trafficked persons. | 115 (71.4) | 515 (71.7) | 0.9232 |
| My current institution has trained adequately its healthcare providers to care for patients who are trafficked persons. | 21 (13.0) | 32 (4.4) | **0.0001** |
| While working at my current institution, I have encountered a patient whom I suspected or knew was a trafficked person. | 10 (6.2) | 41 (5.6) | 0.7096 |
| Within the last three years, I have attended training (such as an in-person or online course) related to human trafficking and healthcare. | 20 (12.4) | 70 (9.5) | 0.3099 |
| I want to learn more about identification, intervention, and prevention of human trafficking. | 133 (82.1) | 676 (92.2) | **0.0003** |
