# Supplementary material for: Caring for trafficked and unidentified patients in the EHR shadows: Shining a light by sharing the data
Source: PLoS One. 2019 Mar 14;14(3):e0213766. doi: 10.1371/journal.pone.0213766 (PMC6417704; doi:10.1371/journal.pone.0213766)
Supplement: S2 Table — (DOCX) [file pone.0213766.s008.docx]

**S2 Table: Survey Responses by Rural or Non-Rural (Suburban or Urban) Residence**

|  | **Rural**  **N=616** | **Non-Rural**  **N=289** | **Fisher’s Exact Test p-value** |
| --- | --- | --- | --- |
| **Confident of ability, understanding and preparedness, N (%)** |  |  |  |
| I can define “human trafficking.” | 426 (69.2) | 205 (70.9) | 0.6416 |
| I can identify multiple types of human trafficking. | 232 (37.7) | 121 (41.9) | 0.2427 |
| I know where human trafficking occurs. | 150 (24.5) | 86 (30) | 0.0881 |
| I am aware of the extent of human trafficking occurring in my state. | 93 (15.2) | 44 (15.2) | 1.0000 |
| I am aware of the extent of human trafficking occurring worldwide. | 224 (36.5) | 93 (32.3) | 0.2316 |
| I understand the physical health consequences of human trafficking. | 342 (55.7) | 163 (56.8) | 0.7736 |
| I understand the psychological health consequences of human trafficking. | 363 (59.4) | 177 (61.5) | 0.6095 |
| I know the warning signs or indicators that a patient is a trafficked person. | 76 (12.4) | 38 (13.3) | 0.7468 |
| I know how to communicate effectively with a patient suspected of being a trafficked person. | 54 (8.8) | 29 (10.1) | 0.5370 |
| I know how to provide trauma-informed medical care for a patient suspected of being a trafficked person. | 81 (13.2) | 38 (13.2) | 1.0000 |
| I know how to provide culturally-sensitive medical care for a patient suspected of being a trafficked person. | 115 (18.7) | 68 (23.6) | 0.0916 |
| I know where trafficked persons can obtain housing assistance. | 46 (7.5) | 22 (7.6) | 1.0000 |
| I know where trafficked persons can obtain legal assistance. | 40 (6.5) | 22 (7.6) | 0.5726 |
| I know where trafficked persons can obtain immigration assistance. | 20 (3.3) | 14 (4.9) | 0.2614 |
| I know where trafficked persons can obtain employment assistance. | 30 (4.9) | 20 (6.9) | 0.2157 |
| I know where trafficked persons can obtain food assistance. | 72 (11.7) | 39 (13.6) | 0.4463 |
| I know how to refer trafficked persons to non-medical services (such as housing, legal, immigration, employment, and food assistance resources). | 61 (10) | 28 (9.8) | 1.0000 |
| I understand the medical record documentation issues related to caring for a patient suspected of being a trafficked person. | 43 (7) | 23 (8) | 0.5861 |
| I understand the confidentiality issues related to caring for a patient suspected of being a trafficked person. | 233 (37.9) | 121 (42.2) | 0.2417 |
| I understand the law enforcement reporting issues related to caring for a patient suspected of being a trafficked person. | 92 (15) | 46 (15.9) | 0.7663 |
| I know how to ensure my own security and safety as a healthcare provider of a trafficked person. | 101 (16.5) | 48 (16.9) | 0.9233 |
| I know how to ensure my patient’s security and safety when I suspect or know the patient is a trafficked person. | 102 (16.6) | 61 (21.2) | 0.1143 |
| I understand the role of healthcare professionals in the prevention of human trafficking. | 125 (20.4) | 68 (23.7) | 0.2958 |
| **Agree with the following statements, N (%)** |  |  |  |
| Referrals to non-medical services (such as housing, employment, immigration, food, or legal services) are not a healthcare professional’s responsibility. | 69 (11.2) | 31 (10.7) | 0.9096 |
| Human trafficking is not a problem in the geographic area where I work as a healthcare professional. | 118 (19.3) | 66 (23.0) | 0.2149 |
| Continuity of care is an acute problem for trafficked persons. | 553 (90.7) | 256 (89.5) | 0.6286 |
| There should be a specific ICD code for use when a patient is suspected or confirmed as a trafficked person. | 473 (78.6) | 219 (77.9) | 0.8608 |
| The use of biometric tools (like palm readers, fingerprinting, and retinal or iris scans) would improve patient safety. | 447 (73.9) | 193 (68.7) | 0.1255 |
| The use of DNA identifiers (or other biomarkers) would improve the continuity of care for trafficked persons. | 441 (73.0) | 198 (70.7) | 0.5182 |
| My current institution has trained adequately its healthcare providers to care for patients who are trafficked persons. | 31 (5.1) | 20 (7.0) | 0.2797 |
| While working at my current institution, I have encountered a patient whom I suspected or knew was a trafficked person. | 37 (6.0) | 14 (4.9) | 0.5387 |
| Within the last three years, I have attended training (such as an in-person or online course) related to human trafficking and healthcare. | 54 (8.8) | 36 (12.5) | 0.0962 |
| I want to learn more about identification, intervention, and prevention of human trafficking. | 549 (89.9) | 263 (91.0) | 0.6322 |
