# Supplementary material for: Caring for trafficked and unidentified patients in the EHR shadows: Shining a light by sharing the data
Source: PLoS One. 2019 Mar 14;14(3):e0213766. doi: 10.1371/journal.pone.0213766 (PMC6417704; doi:10.1371/journal.pone.0213766)
Supplement: S3 Table — (DOCX) [file pone.0213766.s009.docx]

**S3 Table. Survey Responses by Race/Ethnicity**

|  | **White**  **N=808** | **Non-White**  **N=164** | **Fisher’s Exact Test p-value** |
| --- | --- | --- | --- |
| **Confident of ability, understanding and preparedness N (%)** |  |  |  |
| I can define “human trafficking.” | 562 (69.6) | 112 (68.3) | 0.7807 |
| I can identify multiple types of human trafficking. | 306 (37.9) | 74 (45.4) | 0.0789 |
| I know where human trafficking occurs. | 210 (26.1) | 40 (24.8) | 0.7684 |
| I am aware of the extent of human trafficking occurring in my state. | 123 (15.3) | 21 (13) | 0.5447 |
| I am aware of the extent of human trafficking occurring worldwide. | 276 (34.3) | 60 (37.3) | 0.4697 |
| I understand the physical health consequences of human trafficking. | 445 (55.3) | 94 (57.7) | 0.6048 |
| I understand the psychological health consequences of human trafficking. | 478 (59.5) | 99 (61.1) | 0.7262 |
| I know the warning signs or indicators that a patient is a trafficked person. | 98 (12.2) | 21 (13) | 0.7928 |
| I know how to communicate effectively with a patient suspected of being a trafficked person. | 73 (9.1) | 15 (9.3) | 0.8817 |
| I know how to provide trauma-informed medical care for a patient suspected of being a trafficked person. | 103 (12.8) | 22 (13.8) | 0.7005 |
| I know how to provide culturally-sensitive medical care for a patient suspected of being a trafficked person. | 156 (19.3) | 36 (22.2) | 0.3896 |
| I know where trafficked persons can obtain housing assistance. - Confident | 60 (7.5) | 11 (6.8) | 0.8697 |
| I know where trafficked persons can obtain legal assistance. | 57 (7.1) | 7 (4.3) | 0.2284 |
| I know where trafficked persons can obtain immigration assistance. | 30 (3.7) | 5 (3.1) | 0.8207 |
| I know where trafficked persons can obtain employment assistance. | 46 (5.7) | 7 (4.3) | 0.5733 |
| I know where trafficked persons can obtain food assistance. | 98 (12.2) | 21 (13) | 0.7936 |
| I know how to refer trafficked persons to non-medical services (such as housing, legal, immigration, employment, and food assistance resources). | 81 (10.1) | 13 (8.3) | 0.5586 |
| I understand the medical record documentation issues related to caring for a patient suspected of being a trafficked person. | 54 (6.7) | 18 (11.2) | 0.0684 |
| I understand the confidentiality issues related to caring for a patient suspected of being a trafficked person. | 313 (38.9) | 66 (40.7) | 0.6605 |
| I understand the law enforcement reporting issues related to caring for a patient suspected of being a trafficked person. | 124 (15.4) | 23 (14.3) | 0.8103 |
| I know how to ensure my own security and safety as a healthcare provider of a trafficked person. | 132 (16.5) | 27 (16.7) | 1.0000 |
| I know how to ensure my patient’s security and safety when I suspect or know the patient is a trafficked person. | 146 (18.1) | 26 (16) | 0.5747 |
| I understand the role of healthcare professionals in the prevention of human trafficking. | 168 (20.9) | 34 (21.3) | 0.9156 |
| **Agree with the following statements, N (%)** |  |  |  |
| Referrals to non-medical services (such as housing, employment, immigration, food, or legal services) are not a healthcare professional’s responsibility. | 78 (9.7) | 29 (23.6) | **<0.0001** |
| Human trafficking is not a problem in the geographic area where I work as a healthcare professional. | 160 (19.9) | 32 (26.4) | 0.1175 |
| Continuity of care is an acute problem for trafficked persons. | 721 (90.1) | 110 (90.9) | 0.8708 |
| There should be a specific ICD code for use when a patient is suspected or confirmed as a trafficked person. | 624 (79.2) | 85 (70.2) | **0.0335** |
| The use of biometric tools (like palm readers, fingerprinting, and retinal or iris scans) would improve patient safety. | 578 (73.2) | 78 (64.5) | 0.0507 |
| The use of DNA identifiers (or other biomarkers) would improve the continuity of care for trafficked persons. | 570 (72.3) | 82 (67.2) | 0.2802 |
| My current institution has trained adequately its healthcare providers to care for patients who are trafficked persons. | 42 (5.3) | 13 (10.7) | **0.0237** |
| While working at my current institution, I have encountered a patient whom I suspected or knew was a trafficked person. | 47 (5.8) | 6 (5.0) | 0.8354 |
| Within the last three years, I have attended training (such as an in-person or online course) related to human trafficking and healthcare. | 82 (10.2) | 11 (9.0) | 0.8713 |
| I want to learn more about identification, intervention, and prevention of human trafficking. | 727 (90.5) | 107 (87.0) | 0.2554 |
