# Supplementary material for: Caring for trafficked and unidentified patients in the EHR shadows: Shining a light by sharing the data
Source: PLoS One. 2019 Mar 14;14(3):e0213766. doi: 10.1371/journal.pone.0213766 (PMC6417704; doi:10.1371/journal.pone.0213766)
Supplement: S4 Table — (DOCX) [file pone.0213766.s010.docx]

**S4 Table. Survey Responses by Gender Identity**

|  | **Man**  **N=166** | **Woman**  **N=737** | **Fisher’s Exact Test p-value** |
| --- | --- | --- | --- |
| **Confident of ability, understanding and preparedness N (%)** |  |  |  |
| I can define “human trafficking.” | 112 (67.5) | 518 (70.3) | 0.5127 |
| I can identify multiple types of human trafficking. | 76 (45.8) | 275 (37.4) | 0.0523 |
| I know where human trafficking occurs. | 43 (25.9) | 192 (26.3) | 1.0000 |
| I am aware of the extent of human trafficking occurring in my state. | 21 (12.8) | 112 (15.3) | 0.4674 |
| I am aware of the extent of human trafficking occurring worldwide. | 57 (34.5) | 255 (34.7) | 1.0000 |
| I understand the physical health consequences of human trafficking. | 86 (52.4) | 417 (56.7) | 0.3390 |
| I understand the psychological health consequences of human trafficking. | 98 (59) | 444 (60.7) | 0.7253 |
| I know the warning signs or indicators that a patient is a trafficked person. | 23 (14) | 88 (12) | 0.5113 |
| I know how to communicate effectively with a patient suspected of being a trafficked person. | 16 (9.7) | 66 (9) | 0.7656 |
| I know how to provide trauma-informed medical care for a patient suspected of being a trafficked person. | 23 (13.9) | 94 (12.8) | 0.7022 |
| I know how to provide culturally-sensitive medical care for a patient suspected of being a trafficked person. | 28 (16.9) | 152 (20.7) | 0.2847 |
| I know where trafficked persons can obtain housing assistance. - Confident | 14 (8.4) | 52 (7.1) | 0.5132 |
| I know where trafficked persons can obtain legal assistance. | 15 (9) | 46 (6.3) | 0.2295 |
| I know where trafficked persons can obtain immigration assistance. | 11 (6.6) | 22 (3) | **0.0365** |
| I know where trafficked persons can obtain employment assistance. | 10 (6) | 39 (5.3) | 0.7056 |
| I know where trafficked persons can obtain food assistance. | 19 (11.4) | 91 (12.4) | 0.7942 |
| I know how to refer trafficked persons to non-medical services (such as housing, legal, immigration, employment, and food assistance resources). | 19 (11.5) | 69 (9.5) | 0.4692 |
| I understand the medical record documentation issues related to caring for a patient suspected of being a trafficked person. | 18 (10.9) | 48 (6.5) | 0.0673 |
| I understand the confidentiality issues related to caring for a patient suspected of being a trafficked person. | 56 (33.7) | 296 (40.4) | 0.1342 |
| I understand the law enforcement reporting issues related to caring for a patient suspected of being a trafficked person. | 31 (18.8) | 108 (14.7) | 0.1910 |
| I know how to ensure my own security and safety as a healthcare provider of a trafficked person. | 35 (21.1) | 113 (15.5) | 0.1046 |
| I know how to ensure my patient’s security and safety when I suspect or know the patient is a trafficked person. | 34 (20.5) | 129 (17.6) | 0.3738 |
| I understand the role of healthcare professionals in the prevention of human trafficking. | 32 (19.4) | 160 (21.9) | 0.5293 |
| **Agree with the following statements, N (%)** |  |  |  |
| Referrals to non-medical services (such as housing, employment, immigration, food, or legal services) are not a healthcare professional’s responsibility. | 43 (25.9) | 55 (7.5) | **<0.0001** |
| Human trafficking is not a problem in the geographic area where I work as a healthcare professional. | 48 (28.9) | 132 (18.1) | **0.0025** |
| Continuity of care is an acute problem for trafficked persons. | 154 (92.8) | 653 (89.7) | 0.2493 |
| There should be a specific ICD code for use when a patient is suspected or confirmed as a trafficked person. | 120 (73.2) | 575 (80.2) | 0.0559 |
| The use of biometric tools (like palm readers, fingerprinting, and retinal or iris scans) would improve patient safety. | 125 (75.3) | 516 (71.8) | 0.3868 |
| The use of DNA identifiers (or other biomarkers) would improve the continuity of care for trafficked persons. | 122 (73.5) | 516 (72) | 0.7730 |
| My current institution has trained adequately its healthcare providers to care for patients who are trafficked persons. | 16 (9.8) | 34 (4.7) | **0.0146** |
| While working at my current institution, I have encountered a patient whom I suspected or knew was a trafficked person. | 10 (6.1) | 41 (5.6) | 0.8521 |
| Within the last three years, I have attended training (such as an in-person or online course) related to human trafficking and healthcare. | 17 (10.3) | 73 (9.9) | 0.8861 |
| I want to learn more about identification, intervention, and prevention of human trafficking. | 139 (83.7) | 674 (92.1) | **0.0018** |
