# Supplementary material for: Caring for trafficked and unidentified patients in the EHR shadows: Shining a light by sharing the data
Source: PLoS One. 2019 Mar 14;14(3):e0213766. doi: 10.1371/journal.pone.0213766 (PMC6417704; doi:10.1371/journal.pone.0213766)
Supplement: S5 Table — (DOCX) [file pone.0213766.s011.docx]

**S5 Table. Survey Responses by Department**

|  | **Emergency** | **OB/GYN** | **Psychiatry** | **Pediatrics** | **Chi-Square**  **p-value** |
| --- | --- | --- | --- | --- | --- |
| **Confident of ability, understanding and preparedness N (%)** |  |  |  |  |  |
| I can define “human trafficking.” | 50 (67.6) | 34 (68) | 19 (73.1) | 53 (77.9) | 0.5162 |
| I can identify multiple types of human trafficking. | 28 (37.8) | 14 (28) | 14 (53.8) | 23 (33.8) | 0.1570 |
| I know where human trafficking occurs. | 26 (35.6) | 10 (20) | 9 (34.6) | 17 (25) | 0.2154 |
| I am aware of the extent of human trafficking occurring in my state. | 18 (24.3) | 5 (10.2) | 3 (11.5) | 12 (17.6) | 0.1845 |
| I am aware of the extent of human trafficking occurring worldwide. | 31 (42.5) | 9 (18) | 12 (46.2) | 27 (39.7) | **0.0198** |
| I understand the physical health consequences of human trafficking. | 42 (56.8) | 26 (52) | 13 (52) | 40 (59.7) | 0.8310 |
| I understand the psychological health consequences of human trafficking. | 43 (58.1) | 26 (52) | 18 (69.2) | 42 (63.6) | 0.4373 |
| I know the warning signs or indicators that a patient is a trafficked person. | 14 (18.9) | 3 (6) | 5 (20) | 8 (11.9) | 0.1604 |
| I know how to communicate effectively with a patient suspected of being a trafficked person. | 11 (14.9) | 4 (8) | 4 (16) | 3 (4.5) | 0.1484 |
| I know how to provide trauma-informed medical care for a patient suspected of being a trafficked person. | 20 (27) | 3 (6) | 6 (23.1) | 12 (17.6) | **0.0291** |
| I know how to provide culturally-sensitive medical care for a patient suspected of being a trafficked person. | 19 (25.7) | 11 (22) | 6 (23.1) | 15 (22.1) | 0.9527 |
| I know where trafficked persons can obtain housing assistance. - Confident | 9 (12.2) | 3 (6) | 2 (7.7) | 6 (9) | 0.6908 |
| I know where trafficked persons can obtain legal assistance. | 8 (10.8) | 3 (6) | 3 (11.5) | 3 (4.4) | 0.4319 |
| I know where trafficked persons can obtain immigration assistance. | 3 (4.1) | 2 (4) | 1 (3.8) | 3 (4.4) | 0.9991 |
| I know where trafficked persons can obtain employment assistance. | 3 (4.1) | 4 (8) | 3 (11.5) | 3 (4.4) | 0.4603 |
| I know where trafficked persons can obtain food assistance. | 10 (13.5) | 6 (12.5) | 4 (15.4) | 6 (8.8) | 0.7770 |
| I know how to refer trafficked persons to non-medical services (such as housing, legal, immigration, employment, and food assistance resources). | 11 (14.9) | 4 (8.2) | 2 (7.7) | 7 (10.3) | 0.6047 |
| I understand the medical record documentation issues related to caring for a patient suspected of being a trafficked person. | 10 (13.5) | 3 (6) | 3 (11.5) | 4 (5.9) | 0.3427 |
| I understand the confidentiality issues related to caring for a patient suspected of being a trafficked person. | 39 (52.7) | 11 (22.4) | 8 (30.8) | 18 (26.9) | **0.0013** |
| I understand the law enforcement reporting issues related to caring for a patient suspected of being a trafficked person. | 19 (25.7) | 5 (10) | 3 (11.5) | 9 (13.2) | 0.0706 |
| I know how to ensure my own security and safety as a healthcare provider of a trafficked person. | 18 (24.7) | 6 (12.2) | 5 (19.2) | 10 (14.7) | 0.2829 |
| I know how to ensure my patient’s security and safety when I suspect or know the patient is a trafficked person. | 19 (26) | 3 (6) | 9 (36) | 7 (10.3) | **0.0009** |
| I understand the role of healthcare professionals in the prevention of human trafficking. | 21 (28.4) | 9 (18.4) | 7 (26.9) | 14 (20.6) | 0.5344 |
| **Agree with the following statements,**  **N (%)** |  |  |  |  |  |
| Referrals to non-medical services (such as housing, employment, immigration, food, or legal services) are not a healthcare professional’s responsibility. | 12 (16.2) | 7 (14) | 2 (7.7) | 5 (7.4) | 0.3478 |
| Human trafficking is not a problem in the geographic area where I work as a healthcare professional. | 12 (16.2) | 8 (16) | 5 (19.2) | 13 (19.4) | 0.9435 |
| Continuity of care is an acute problem for trafficked persons. | 62 (83.8) | 46 (92) | 24 (92.3) | 65 (95.6) | 0.1078 |
| There should be a specific ICD code for use when a patient is suspected or confirmed as a trafficked person. | 50 (70.4) | 41 (85.4) | 17 (65.4) | 58 (85.3) | **0.0382** |
| The use of biometric tools (like palm readers, fingerprinting, and retinal or iris scans) would improve patient safety. | 52 (72.2) | 38 (77.6) | 21 (80.8) | 44 (64.7) | 0.3152 |
| The use of DNA identifiers (or other biomarkers) would improve the continuity of care for trafficked persons. | 48 (66.7) | 39 (79.6) | 18 (69.2) | 45 (66.2) | 0.3932 |
| While working at my current institution, I have encountered a patient whom I suspected or knew was a trafficked person. | 6 (8.1) | 3 (6) | 0 (0) | 3 (4.5) | 0.4528 |
| My current institution has trained adequately its healthcare providers to care for patients who are trafficked persons. | 14 (18.9) | 4 (8) | 3 (11.5) | 6 (8.8) | 0.2027 |
| Within the last three years, I have attended training (such as an in-person or online course) related to human trafficking and healthcare. | 22 (29.7) | 2 (4) | 4 (15.4) | 11 (16.2) | **0.0031** |
| I want to learn more about identification, intervention, and prevention of human trafficking. | 65 (87.8) | 49 (98) | 25 (96.2) | 58 (86.6) | 0.1023 |
