# Supplementary material for: Caring for trafficked and unidentified patients in the EHR shadows: Shining a light by sharing the data
Source: PLoS One. 2019 Mar 14;14(3):e0213766. doi: 10.1371/journal.pone.0213766 (PMC6417704; doi:10.1371/journal.pone.0213766)
Supplement: S6 Table — (DOCX) [file pone.0213766.s012.docx]

**S6 Table. Survey Responses by Years in the Healthcare Profession**

|  | **Fewer than 10 years** | **10-19 years** | **20-29 years** | **30 years or more** | **Chi-Square**  **p-value** |
| --- | --- | --- | --- | --- | --- |
| **Confident of ability, understanding and preparedness N (%)** |  |  |  |  |  |
| I can define “human trafficking.” | 208 (74) | 148 (69.2) | 114 (67.1) | 164 (68.3) | 0.3538 |
| I can identify multiple types of human trafficking. | 103 (36.7) | 85 (39.7) | 63 (37.1) | 102 (42.7) | 0.5082 |
| I know where human trafficking occurs. | 89 (31.7) | 59 (27.6) | 32 (19.3) | 57 (23.8) | **0.0253** |
| I am aware of the extent of human trafficking occurring in my state. | 47 (16.8) | 32 (15) | 18 (10.7) | 36 (15.2) | 0.3591 |
| I am aware of the extent of human trafficking occurring worldwide. | 92 (33.1) | 81 (37.9) | 49 (29) | 91 (37.9) | 0.1890 |
| I understand the physical health consequences of human trafficking. | 162 (57.7) | 126 (59.2) | 85 (50.6) | 131 (54.6) | 0.3406 |
| I understand the psychological health consequences of human trafficking. | 177 (63.2) | 131 (61.8) | 92 (54.8) | 139 (58.2) | 0.2903 |
| I know the warning signs or indicators that a patient is a trafficked person. | 41 (14.7) | 23 (10.7) | 16 (9.4) | 33 (13.9) | 0.2950 |
| I know how to communicate effectively with a patient suspected of being a trafficked person. | 28 (10.1) | 23 (10.7) | 13 (7.6) | 19 (8) | 0.6204 |
| I know how to provide trauma-informed medical care for a patient suspected of being a trafficked person. | 36 (12.8) | 30 (14.1) | 26 (15.4) | 28 (11.7) | 0.7138 |
| I know how to provide culturally-sensitive medical care for a patient suspected of being a trafficked person. | 66 (23.5) | 41 (19.2) | 40 (23.5) | 34 (14.2) | **0.0363** |
| I know where trafficked persons can obtain housing assistance. - Confident | 22 (7.8) | 16 (7.5) | 13 (7.6) | 18 (7.6) | 0.9992 |
| I know where trafficked persons can obtain legal assistance. | 24 (8.5) | 12 (5.6) | 11 (6.5) | 13 (5.4) | 0.4637 |
| I know where trafficked persons can obtain immigration assistance. | 15 (5.3) | 5 (2.3) | 6 (3.5) | 7 (2.9) | 0.2994 |
| I know where trafficked persons can obtain employment assistance. | 19 (6.8) | 9 (4.2) | 8 (4.8) | 13 (5.4) | 0.6210 |
| I know where trafficked persons can obtain food assistance. | 33 (11.8) | 20 (9.3) | 21 (12.4) | 34 (14.3) | 0.4502 |
| I know how to refer trafficked persons to non-medical services (such as housing, legal, immigration, employment, and food assistance resources). | 30 (10.7) | 18 (8.5) | 17 (10.1) | 24 (10.3) | 0.8605 |
| I understand the medical record documentation issues related to caring for a patient suspected of being a trafficked person. | 18 (6.4) | 19 (8.9) | 15 (8.8) | 16 (6.8) | 0.6409 |
| I understand the confidentiality issues related to caring for a patient suspected of being a trafficked person. | 102 (36.4) | 84 (39.3) | 68 (40.5) | 101 (42.3) | 0.5855 |
| I understand the law enforcement reporting issues related to caring for a patient suspected of being a trafficked person. | 51 (18.3) | 34 (15.9) | 23 (13.6) | 32 (13.3) | 0.3935 |
| I know how to ensure my own security and safety as a healthcare provider of a trafficked person. | 49 (17.8) | 33 (15.4) | 31 (18.3) | 38 (16.1) | 0.8411 |
| I know how to ensure my patient’s security and safety when I suspect or know the patient is a trafficked person. | 53 (18.9) | 41 (19.2) | 34 (20) | 36 (15.1) | 0.5491 |
| I understand the role of healthcare professionals in the prevention of human trafficking. | 70 (25.2) | 55 (25.8) | 28 (16.6) | 38 (16) | **0.0097** |
| **Agree with the following statements,**  **N (%)** |  |  |  |  |  |
| Referrals to non-medical services (such as housing, employment, immigration, food, or legal services) are not a healthcare professional’s responsibility. | 31 (11) | 22 (10.3) | 24 (14.2) | 27 (11.3) | 0.6596 |
| Human trafficking is not a problem in the geographic area where I work as a healthcare professional. | 52 (18.6) | 42 (19.7) | 36 (21.4) | 54 (22.7) | 0.6803 |
| Continuity of care is an acute problem for trafficked persons. | 244 (86.8) | 190 (89.2) | 158 (95.8) | 216 (91.1) | **0.0201** |
| There should be a specific ICD code for use when a patient is suspected or confirmed as a trafficked person. | 229 (82.4) | 161 (75.9) | 130 (79.3) | 173 (75.5) | 0.2095 |
| The use of biometric tools (like palm readers, fingerprinting, and retinal or iris scans) would improve patient safety. | 193 (69.2) | 151 (71.6) | 127 (77) | 172 (74.5) | 0.2908 |
| The use of DNA identifiers (or other biomarkers) would improve the continuity of care for trafficked persons. | 205 (73.5) | 141 (66.8) | 124 (75.6) | 171 (74.3) | 0.1924 |
| While working at my current institution, I have encountered a patient whom I suspected or knew was a trafficked person. | 19 (6.8) | 12 (5.7) | 7 (4.2) | 15 (6.3) | 0.7081 |
| My current institution has trained adequately its healthcare providers to care for patients who are trafficked persons. | 17 (6.1) | 11 (5.1) | 14 (8.3) | 10 (4.2) | 0.3424 |
| Within the last three years, I have attended training (such as an in-person or online course) related to human trafficking and healthcare. | 26 (9.3) | 23 (10.7) | 18 (10.7) | 23 (9.6) | 0.9351 |
| I want to learn more about identification, intervention, and prevention of human trafficking. | 257 (91.5) | 189 (89.2) | 152 (90.5) | 215 (90) | 0.8519 |
