# Supplementary material for: Caring for trafficked and unidentified patients in the EHR shadows: Shining a light by sharing the data
Source: PLoS One. 2019 Mar 14;14(3):e0213766. doi: 10.1371/journal.pone.0213766 (PMC6417704; doi:10.1371/journal.pone.0213766)
Supplement: S7 Table — (DOCX) [file pone.0213766.s013.docx]

**S7a Table. Survey Responses to “While working at my current institution, I have encountered a patient whom I suspected or knew was a trafficked person” by 3-Digit Work Zip Code**

| Work Zip Code | While working at my current institution, I have encountered a patient whom I suspected or knew was a trafficked person. | | Chi-Square Test  p-value |
| --- | --- | --- | --- |
|  | Agree  N=45 | Disagree  N=805 |  |
| 166** | 1 (2.2) | 2 (0.25) | 0.0364 |
| 168** | 0 (0) | 21 (2.6) |  |
| 170** | 16 (35.6) | 153 (19.0) |  |
| 171** | 1 (2.2) | 5 (0.62) |  |
| 177** | 0 (0) | 12 (1.5) |  |
| 178** | 23 (51.1) | 402 (49.9) |  |
| 179** | 0 (0) | 14 (1.7) |  |
| 180** | 0 (0) | 1 (0.12) |  |
| 184** | 1 (2.2) | 8 (0.99) |  |
| 185** | 1 (2.2) | 55 (6.8) |  |
| 186** | 0 (0) | 11 (1.4) |  |
| 187** | 2 (4.4) | 121 (15.0) |  |

**S7b Table. Survey Responses to “While working at my current institution, I have encountered a patient whom I suspected or knew was a trafficked person” by 3-Digit Work Zip Code (state capitol region vs. all others)**

| Work Zip Code | While working at my current institution, I have encountered a patient whom I suspected or knew was a trafficked person. | | Fisher’s Exact Test  p-value |
| --- | --- | --- | --- |
|  | Agree  N=45 | Disagree  N=805 |  |
| 170** | 16 (35.6) | 153 (19.0) | 0.0114 |
| All Other Zip Codes | 29 (64.4) | 652 (81.0) |  |

**S7c Table. Survey Responses to “While working at my current institution, I have encountered a patient whom I suspected or knew was a trafficked person” by 3-Digit Work Zip Code (with hospitals vs. without hospitals)**

| Work Zip Code | While working at my current institution, I have encountered a patient whom I suspected or knew was a trafficked person. | | Fisher’s Exact Test  p-value |
| --- | --- | --- | --- |
|  | Agree  N=45 | Disagree  N=805 |  |
| Hospital Zip Codes | 41 (91.1) | 687 (85.3) | 0.3828 |
| All Other Zip Codes | 4 (8.9) | 118 (14.7) |  |
