# Supplementary material for: Caring for trafficked and unidentified patients in the EHR shadows: Shining a light by sharing the data
Source: PLoS One. 2019 Mar 14;14(3):e0213766. doi: 10.1371/journal.pone.0213766 (PMC6417704; doi:10.1371/journal.pone.0213766)
Supplement: S8 Table — (DOCX) [file pone.0213766.s014.docx]

**S8 Table. Survey Responses by Confidence in “I can define human trafficking.”**

Comparison of responses by those reporting Confident and Very Confident with those reporting Not Confident and Hesitant.

|  | **Confident** | **Hesitant** | **Fisher’s Exact Test p-value** |
| --- | --- | --- | --- |
| **Confident of ability, understanding and preparedness N (%)** |  |  |  |
| I can identify multiple types of human trafficking. | 374 (55.6) | 6 (2.0) | **<0.0001** |
| I know where human trafficking occurs. | 240 (35.9) | 10 (3.4) | **<0.0001** |
| I am aware of the extent of human trafficking occurring in my state. | 125 (18.7) | 19 (6.4) | **<0.0001** |
| I am aware of the extent of human trafficking occurring worldwide. | 293 (43.7) | 43 (14.6) | **<0.0001** |
| I understand the physical health consequences of human trafficking. | 466 (69.3) | 73 (24.7) | **<0.0001** |
| I understand the psychological health consequences of human trafficking. | 488 (72.7) | 89 (30.3) | **<0.0001** |
| I know the warning signs or indicators that a patient is a trafficked person. | 117 (17.5) | 2 (0.7) | **<0.0001** |
| I know how to communicate effectively with a patient suspected of being a trafficked person. | 85 (12.7) | 3 (1.0) | **<0.0001** |
| I know how to provide trauma-informed medical care for a patient suspected of being a trafficked person. | 114 (17.0) | 11 (3.7) | **<0.0001** |
| I know how to provide culturally-sensitive medical care for a patient suspected of being a trafficked person. | 174 (25.9) | 18 (6.1) | **<0.0001** |
| I know where trafficked persons can obtain housing assistance. - Confident | 64 (9.5) | 7 (2.4) | **<0.0001** |
| I know where trafficked persons can obtain legal assistance. | 56 (8.3) | 8 (2.7) | **0.0007** |
| I know where trafficked persons can obtain immigration assistance. | 32 (4.8) | 3 (1.0) | **0.0025** |
| I know where trafficked persons can obtain employment assistance. | 48 (7.2) | 5 (1.7) | **0.0003** |
| I know where trafficked persons can obtain food assistance. | 106 (15.8) | 13 (4.4) | **<0.0001** |
| I know how to refer trafficked persons to non-medical services (such as housing, legal, immigration, employment, and food assistance resources). | 83 (12.5) | 11 (3.8) | **<0.0001** |
| I understand the medical record documentation issues related to caring for a patient suspected of being a trafficked person. | 68 (10.1) | 4 (1.4) | **<0.0001** |
| I understand the confidentiality issues related to caring for a patient suspected of being a trafficked person. | 322 (48.1) | 57 (19.3) | **<0.0001** |
| I understand the law enforcement reporting issues related to caring for a patient suspected of being a trafficked person. | 133 (19.9) | 14 (4.7) | **<0.0001** |
| I know how to ensure my own security and safety as a healthcare provider of a trafficked person. | 138 (20.8) | 21 (7.1) | **<0.0001** |
| I know how to ensure my patient’s security and safety when I suspect or know the patient is a trafficked person. | 150 (22.4) | 22 (7.4) | **<0.0001** |
| I understand the role of healthcare professionals in the prevention of human trafficking. | 186 (27.8) | 16 (5.4) | **<0.0001** |
| **Agree with the following statements, N (%)** |  |  |  |
| Referrals to non-medical services (such as housing, employment, immigration, food, or legal services) are not a healthcare professional’s responsibility. | 66 (10.2) | 41 (14.5) | 0.0582 |
| Human trafficking is not a problem in the geographic area where I work as a healthcare professional. | 114 (17.7) | 78 (28.0) | **0.0006** |
| Continuity of care is an acute problem for trafficked persons. | 579 (90.2) | 252 (90.3) | 1.0000 |
| There should be a specific ICD code for use when a patient is suspected or confirmed as a trafficked person. | 497 (78.4) | 212 (77.1) | 0.6638 |
| The use of biometric tools (like palm readers, fingerprinting, and retinal or iris scans) would improve patient safety. | 454 (71.6) | 202 (72.9) | 0.7484 |
| The use of DNA identifiers (or other biomarkers) would improve the continuity of care for trafficked persons. | 446 (70.2) | 206 (74.9) | 0.1733 |
| My current institution has trained adequately its healthcare providers to care for patients who are trafficked persons. | 43 (6.7) | 12 (4.3) | 0.2241 |
| While working at my current institution, I have encountered a patient whom I suspected or knew was a trafficked person. | 46 (7.1) | 7 (2.5) | **0.0051** |
| Within the last three years, I have attended training (such as an in-person or online course) related to human trafficking and healthcare. | 80 (12.4) | 13 (4.6) | **0.0002** |
| I want to learn more about identification, intervention, and prevention of human trafficking. | 587 (90.7) | 247 (88.5) | 0.3381 |
