# Supplementary material for: Caring for trafficked and unidentified patients in the EHR shadows: Shining a light by sharing the data
Source: PLoS One. 2019 Mar 14;14(3):e0213766. doi: 10.1371/journal.pone.0213766 (PMC6417704; doi:10.1371/journal.pone.0213766)
Supplement: S9 Table — (DOCX) [file pone.0213766.s015.docx]

**S9 Table. Survey Response by Agreement with “While working at my current institution, I have encountered a patient whom I suspected or knew was a trafficked person.”**

Comparison of responses by those reporting Strongly Agree and Agree with those reporting Disagree and Strongly Disagree.

|  | **Agree**  **N=53** | **Disagree**  **N=873** | **Fisher’s Exact Test p-value** |
| --- | --- | --- | --- |
| **Confident of ability, understanding and preparedness N (%)** |  |  |  |
| I can define “human trafficking.” | 46 (86.8) | 601 (68.8) | **0.0051** |
| I can identify multiple types of human trafficking. | 35 (66.0) | 326 (37.4) | **<0.0001** |
| I know where human trafficking occurs. | 28 (52.8) | 212 (24.5) | **<0.0001** |
| I am aware of the extent of human trafficking occurring in my state. | 21 (39.6) | 117 (13.5) | **<0.0001** |
| I am aware of the extent of human trafficking occurring worldwide. | 30 (57.7) | 292 (33.6) | **0.0008** |
| I understand the physical health consequences of human trafficking. | 40 (75.5) | 473 (54.4) | **0.0026** |
| I understand the psychological health consequences of human trafficking. | 39 (73.6) | 510 (58.8) | **0.0426** |
| I know the warning signs or indicators that a patient is a trafficked person. | 22 (41.5) | 94 (10.8) | **<0.0001** |
| I know how to communicate effectively with a patient suspected of being a trafficked person. | 17 (32.1) | 66 (7.6) | **<0.0001** |
| I know how to provide trauma-informed medical care for a patient suspected of being a trafficked person. | 18 (34.0) | 102 (11.7) | **<0.0001** |
| I know how to provide culturally-sensitive medical care for a patient suspected of being a trafficked person. | 21 (39.6) | 162 (18.6) | **0.0006** |
| I know where trafficked persons can obtain housing assistance. - Confident | 16 (30.8) | 53 (6.1) | **<0.0001** |
| I know where trafficked persons can obtain legal assistance. | 16 (30.8) | 46 (5.3) | **<0.0001** |
| I know where trafficked persons can obtain immigration assistance. | 10 (19.2) | 24 (2.8) | **<0.0001** |
| I know where trafficked persons can obtain employment assistance. | 12 (22.6) | 38 (4.4) | **<0.0001** |
| I know where trafficked persons can obtain food assistance. | 21 (39.6) | 89 (10.3) | **<0.0001** |
| I know how to refer trafficked persons to non-medical services (such as housing, legal, immigration, employment, and food assistance resources). | 18 (34.0) | 71 (8.2) | **<0.0001** |
| I understand the medical record documentation issues related to caring for a patient suspected of being a trafficked person. | 14 (26.4) | 55 (6.3) | **<0.0001** |
| I understand the confidentiality issues related to caring for a patient suspected of being a trafficked person. | 33 (62.3) | 326 (37.5) | **0.0004** |
| I understand the law enforcement reporting issues related to caring for a patient suspected of being a trafficked person. | 20 (37.7) | 120 (13.8) | **<0.0001** |
| I know how to ensure my own security and safety as a healthcare provider of a trafficked person. | 22 (41.5) | 130 (15.1) | <0.0001 |
| I know how to ensure my patient’s security and safety when I suspect or know the patient is a trafficked person. | 20 (37.7) | 146 (16.8) | 0.0006 |
| I understand the role of healthcare professionals in the prevention of human trafficking. | 29 (54.7) | 167 (19.3) | <0.0001 |
| **Agree with the following statements, N (%)** |  |  |  |
| Referrals to non-medical services (such as housing, employment, immigration, food, or legal services) are not a healthcare professional’s responsibility. | 9 (17) | 97 (11.1) | 0.1852 |
| Human trafficking is not a problem in the geographic area where I work as a healthcare professional. | 11 (20.8) | 180 (20.7) | 1.0000 |
| Continuity of care is an acute problem for trafficked persons. | 46 (86.8) | 783 (90.4) | 0.3462 |
| There should be a specific ICD code for use when a patient is suspected or confirmed as a trafficked person. | 38 (73.1) | 670 (78.4) | 0.3885 |
| The use of biometric tools (like palm readers, fingerprinting, and retinal or iris scans) would improve patient safety. | 40 (75.5) | 614 (71.7) | 0.6381 |
| The use of DNA identifiers (or other biomarkers) would improve the continuity of care for trafficked persons. | 37 (69.8) | 614 (71.8) | 0.7545 |
| My current institution has trained adequately its healthcare providers to care for patients who are trafficked persons. | 13 (24.5) | 42 (4.9) | **<0.0001** |
| Within the last three years, I have attended training (such as an in-person or online course) related to human trafficking and healthcare. | 24 (45.3) | 69 (7.9) | **<0.0001** |
| I want to learn more about identification, intervention, and prevention of human trafficking. | 49 (92.5) | 783 (89.9) | 0.812 |
